# Supplementary material for: Early Signs of Molecular Defects in iPSC-Derived Neural Stems Cells from Patients with Familial Parkinson’s Disease
Source: Biomolecules. 2022 Jun 23;12(7):876. doi: 10.3390/biom12070876 (PMC9313424; doi:10.3390/biom12070876)
Supplement: Supplementary file 1 [file biomolecules-12-00876-s001.zip › biomolecules-1744156-supplementary.pdf]

Supplementary Methods

RNA sequencing and bioinformatics analysis. Total cellular RNA was extracted from the following samples and sequenced on an Illumina HiSeq sequencer at the European Molecular Biology Laboratory (EMBL): PD and CTR iPSCs differentiated into NPCs (two lines each; PD1/PD2 and CTR1/CTR2). RNA libraries were prepared with the TruSeq RNA Sample Preparation Kit V2 (Illumina). Raw RNA-seq reads were quality checked using FastQC (www.bioinformatics.babraham.ac.uk/projects/fastqc/). Contaminants were detected and removed utilizing a combination of an in-house-developed algorithm and already available tools (5). Following pre-processing, GSNAP spliced aligner (6) was utilized to map the RNA-seq reads against the reference genome (GRCh37/hg19 genome assembly). GSNAP has been appropriately parameterized in order to detect novel and known splice junctions. Differential expression analysis was performed using DESeq (7). Specifically, differential expressed genes with fold change >1 ($p<0.05$) were identified using all expressed genes (>10 raw read counts per gene). Correction for false discovery rate (FDR) was not applied due to the limited number of the resulting differential expressed genes. Heat maps were implemented using R/Bioconductor (8, 9) (Clustering: Pearson'scorrelation coefficient, row-wise scaling: centering and scaling using Z-scores). Enrichment analyses were performed against Gene Ontology10 using the hypergeometric distribution, KEGG and Reactome pathway databases. All gene annotations were derived from Ensembl (10). Heat maps were generated using R programming language in RStudio v1.1.456. All gene annotations were derived from Ensembl (10). Gene Ontology (GO) Enrichment analyses were performed using the Database for Annotation, Visualisation and Integrated Discovery (DAVID) v6.8 (https://david.ncifcrf.gov).

qPCR analysis. Quantitative PCR analyses were carried out in a Light Cycler96 (Roche) Real time PCR detection system using KAPA SYBR FAST qPCR Master Mix (KapaBiosystems). The analysis of expression levels was based on the comparative CT Method ($2^{-\Delta\Delta CT}$ Method) (Livak and Schmittgen, 2001; Schmittgen and Livak, 2008). Briefly, this approach calculates mean C_T values of the target and reference gene (GAPDH) and the values of the reference gene are subtracted from target gene (ΔCt). The biggest ΔCt value is then subtracted from all samples, giving the ΔΔCt. The negative value of this subtraction (-ΔΔCt) is then used as the exponent of 2 and this value represents the fold change of expression. Differences between PD and CTR mRNA levels of selected transcripts from 3 independent differentiation procedures were assessed by two tailed unpaired T-test ($p<0.05$).

Artificial synapse formation assay. To address whether p.A53T-αSyn causes early synaptogenesis defects artificial synapse formation assays (ASFs) were employed as they represent a powerful tool to screen CAMs (Biederer and Scheiffele, 2007). ASFs are co-cultures of non-neuronal cells that present a post-synaptic adhesion neuronal protein on their plasma membrane, with neurons. These proteins are adequate to induce the formation of presynaptic specializatons (Biederer and Scheiffele, 2007). In the present study iPSC-

derived neurons were employed to investigate the synaptic connections between mutant neurons and HEK293T cells overexpressing two of the most well studied postsynaptic molecules,NLGN2 and NLGN4, which belong to the family of neuroligins, a class of four major cell adhesion molecules (Biederer and Scheiffele, 2007). Therefore no additional positive control plasmids were required for this study.

HEK293T (500K cells/well) were plated in 6-well plates and transfected with EGFP (control plasmid, pCMV-EGFP, InVitrogen), and hemagglutinin (HA) tagged NLGN2 (pCMV3-SP-NLNG2-HA, SINO Biological) and NLGN4 (Pcag-HA-NLGN4, Addgene) plasmids using calcium phosphate. Transfected HEK293T cells, were incubated at 37°C for 16-18 hours and cells with transfection efficiency of ~75-80% were dissociated and seeded at a density of 20k cells/well on neurons for 24 hours. Co-cultures were fixed with 4% PFA and stained for Synapsin1 and HA antibodies, to visualize the presynaptic induction of the selected postsynaptic molecules. Artificial contacts were captured by confocal imaging and analyzed by ImageJ software. To semi-automatically quantify the artificial synaptic potency of the two neuroligins, a pipeline based on the co-culture index (C.I.) measurement was used. Briefly, this approach counts the Synapsin1 density that is related to the target HEK293T cells, in a 2 μm radius ring inward and outward from the edge HEK293T, and excludes background Synapsin1 distribution, in a 4 μm radius ring 10 μm away from the HEK293T. HEK293T cells with CI≥1 are considered as positive cells and synaptic formation is measured (Jiang et al., 2017). Data was derived from n=2-3 independent culture systems.

Supplementary References

Livak KJ, Schmittgen TD. 2001. Analysis of relative gene expression data using real-time quantitative PCR and the 2(-Delta Delta C(T)) method. *Methods* 25: 402– 408.

Schmittgen TD, Livak KJ. 2008. Analyzing real-time PCR data by the comparative C_T method. *Nat Protoc* 3: 1101– 1108.

Thomas Biederer and Peter Scheiffele, 2007. Mixed-culture assays for analyzing neuronal synapse formation. *Nat Protoc* 2(3):670-6.

Jiang, W. *et al.* 2017. Identification of Protein Tyrosine Phosphatase Receptor Type O (PTPRO) as a Synaptic Adhesion Molecule that Promotes Synapse Formation. *J Neurosci* 37, 9828-9843.

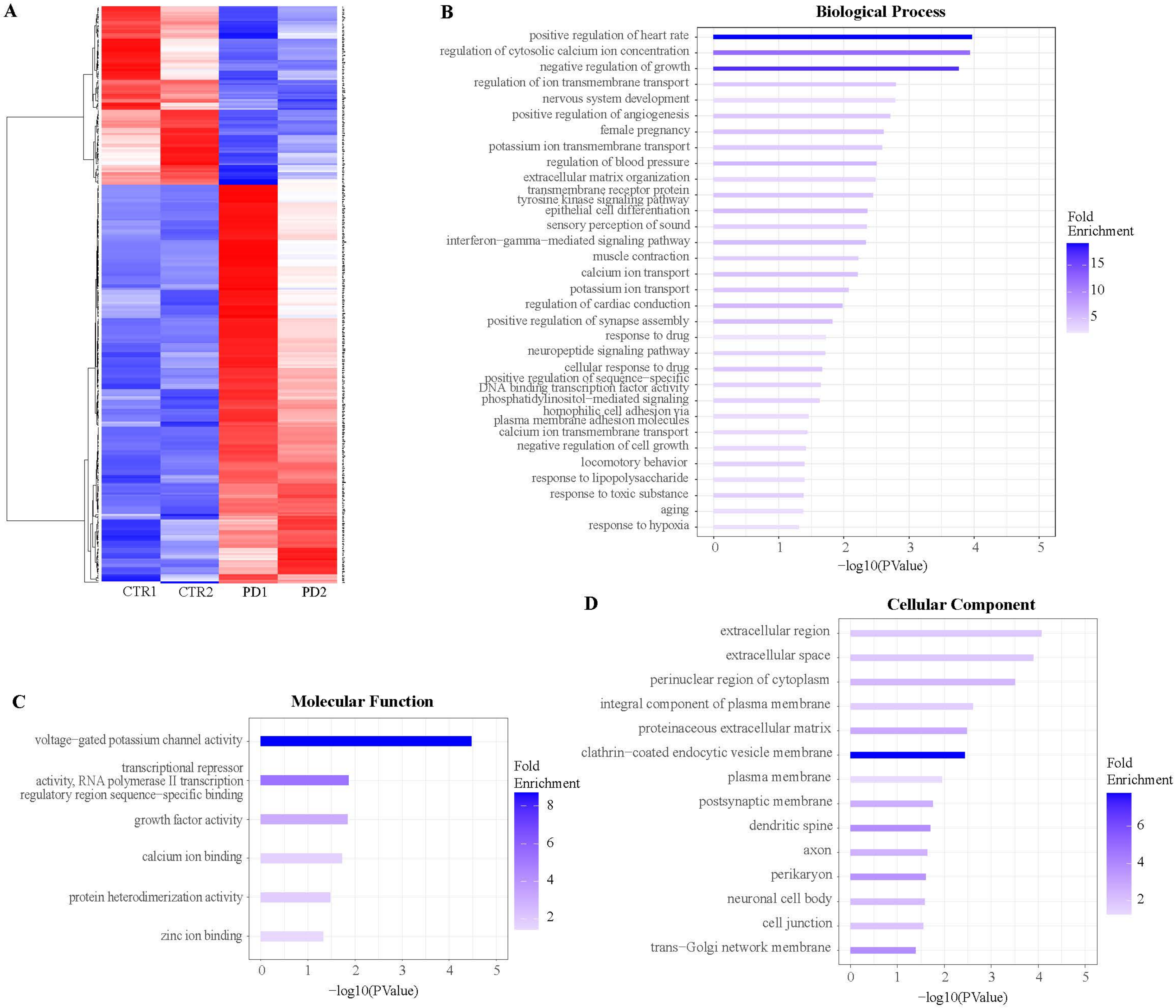

A
CTR1
CTR2
PD1
PD2
B
Biological Process
positive regulation of heart rate
regulation of cytosolic calcium ion concentration
negative regulation of growth
regulation of ion transmembrane transport
nervous system development
positive regulation of angiogenesis
female pregnancy
potassium ion transmembrane transport
regulation of blood pressure
extracellular matrix organization
transmembrane receptor protein tyrosine kinase signaling pathway
epithelial cell differentiation
sensory perception of sound
interferon−gamma−mediated signaling pathway
muscle contraction
calcium ion transport
potassium ion transport
regulation of cardiac conduction
positive regulation of synapse assembly
response to drug
neuropeptide signaling pathway
cellular response to drug
positive regulation of sequence−specific DNA binding transcription factor activity
phosphatidylinositol−mediated signaling
homophilic cell adhesion via plasma membrane adhesion molecules
calcium ion transmembrane transport
negative regulation of cell growth
locomotory behavior
response to lipopolysaccharide
response to toxic substance
aging
response to hypoxia
0 1 2 3 4 5
−log10(PValue)
Fold Enrichment
15
10
5
C
Molecular Function
voltage−gated potassium channel activity
transcriptional repressor activity, RNA polymerase II transcription regulatory region sequence−specific binding
growth factor activity
calcium ion binding
protein heterodimerization activity
zinc ion binding
0 1 2 3 4 5
−log10(PValue)
Fold Enrichment
8
6
4
2
D
Cellular Component
extracellular region
extracellular space
perinuclear region of cytoplasm
integral component of plasma membrane
proteinaceous extracellular matrix
clathrin−coated endocytic vesicle membrane
plasma membrane
postsynaptic membrane
dendritic spine
axon
perikaryon
neuronal cell body
cell junction
trans−Golgi network membrane
0 1 2 3 4 5
−log10(PValue)
Fold Enrichment
6
4
2

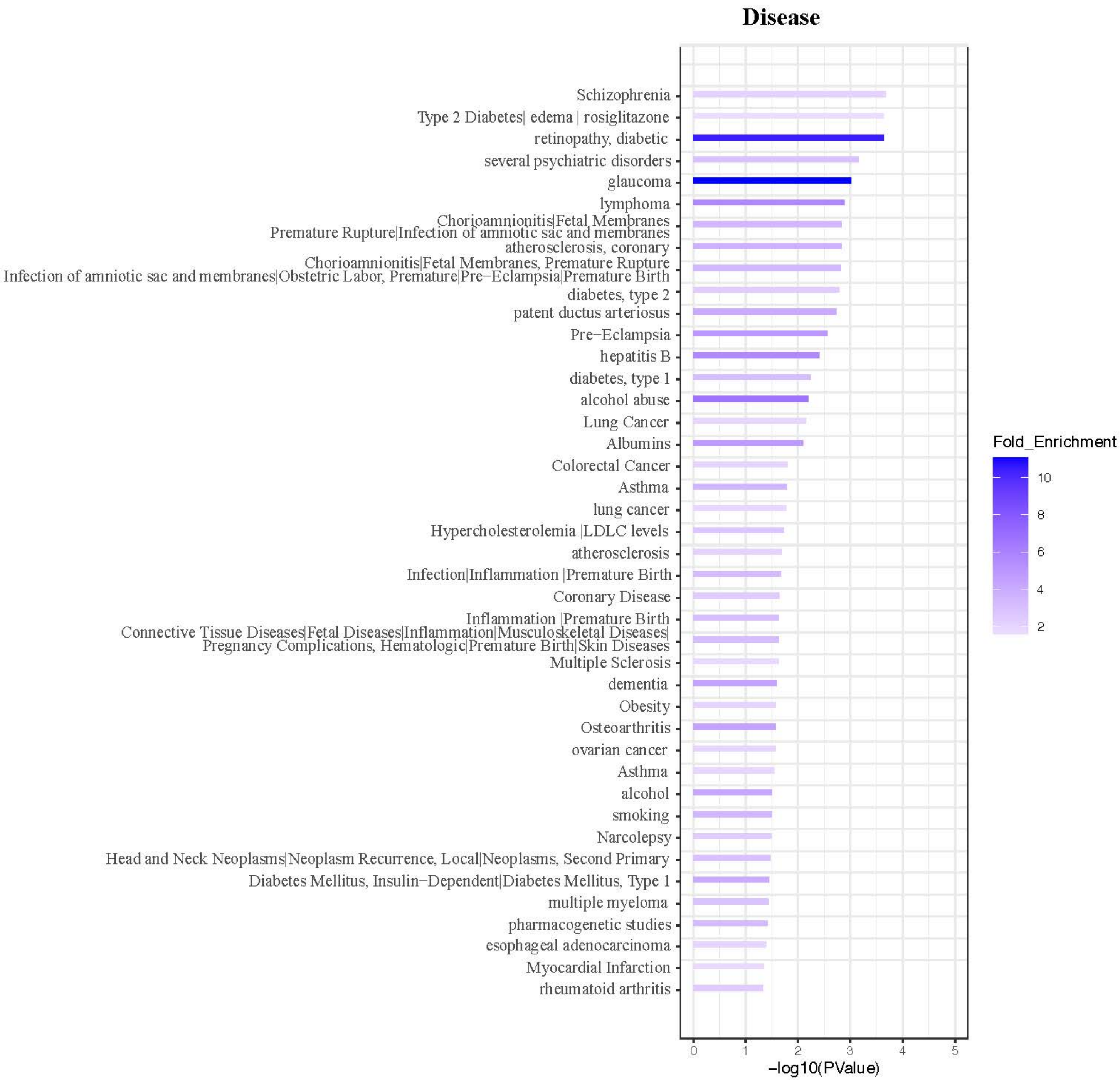

Disease
Schizophrenia
Type 2 Diabetes| edema | rosiglitazone
retinopathy, diabetic
several psychiatric disorders
glaucoma
lymphoma
Chorioamnionitis|Fetal Membranes
Premature Rupture|Infection of amniotic sac and membranes
atherosclerosis, coronary
Chorioamnionitis|Fetal Membranes, Premature Rupture
Infection of amniotic sac and membranes|Obstetric Labor, Premature|Pre−Eclampsia|Premature Birth
diabetes, type 2
patent ductus arteriosus
Pre−Eclampsia
hepatitis B
diabetes, type 1
alcohol abuse
Lung Cancer
Albumins
Colorectal Cancer
Asthma
lung cancer
Hypercholesterolemia |LDLC levels
atherosclerosis
Infection|Inflammation |Premature Birth
Coronary Disease
Inflammation |Premature Birth
Connective Tissue Diseases|Fetal Diseases|Inflammation|Musculoskeletal Diseases|
Pregnancy Complications, Hematologic|Premature Birth|Skin Diseases
Multiple Sclerosis
dementia
Obesity
Osteoarthritis
ovarian cancer
Asthma
alcohol
smoking
Narcolepsy
Head and Neck Neoplasms|Neoplasm Recurrence, Local|Neoplasms, Second Primary
Diabetes Mellitus, Insulin−Dependent|Diabetes Mellitus, Type 1
multiple myeloma
pharmacogenetic studies
esophageal adenocarcinoma
Myocardial Infarction
rheumatoid arthritis
0
1
2
3
4
5
−log10(PValue)
Fold_Enrichment
10
8
6
4
2

Table S1. List of primer sequences

Gene Name	Application	Forward	Reverse
TWY3	qPCR	GGAGGAATAGTGGCTGTAGTTG	CTTCAGCTCCTTTAGCTTCATTTG
CA7	qPCR	AGAAGGCTGGATTGGAGTTATC	AATACGTGAGCCAACTCTACAA
CRYZ	qPCR	AAACCCACGAGACACCATG	CAACCAGCCAATTTCCATTCC
NEUROD1	qPCR	GTTCAGGACCTCTTCCCATTG	AACCCAGATAGCAACATCCAC
NEUROG1	qPCR	GGAGCCTGTAAGGCCAATAA	CATCTGTGATCCCGCTGTAA
FLRT2	qPCR	GGGCCACAGTTTAGTAGGG	CACTAAACAAATCCGATAGGTGG
GABRQ	qPCR	GGTTCCTACATACGCCTGATAC	CAAGAGGTAATAGTGGTGAGGAC
GAPDH	qPCR	CCTCTGACTTCAACAGCGACAC	AGCCAAATTCGTTGTCATACCAG
NESTIN	qPCR	TCCAGGAACGGAAAATCAAG	GCCTCCTCATCCCCTACTTC
NEUROG2	qPCR	TTGCTCAAAGAACTCCAGGG	TCTGCATGTAAGTCACCCAC
PAX6	qPCR	TGTCCAACGGATGTGTGAGT	TTTCCCAAGCAAAGATGGAC
NRK	qPCR	AGCAGTGGCCAACAGTATC	GGTTGCCTGGTCCGTATTTA
FLRT1	qPCR	CTTCAGGCCTCTCCAGATAAAG	GGGTTTGTTCAGGTCATAGGT
NXPH3	qPCR	TCGATCTCCCAGGCTCTTT	GGCGGAGTTTACGATGTATCC
ROBO1	qPCR	TCAGGGAAGGTGGTATGGAT	GTATGCTCTGATGCCGTCTATG
GRID1	qPCR	AGGTCATCATCGAGGAGTCATA	GCTCACCGTAATTGCCTCTAA
SNCA	qPCR	GGAGTGGCCATTCGACGAC	CCTGCTGCTTCTGCCACAC
SYT7	qPCR	TCTCCATCCTCAGCTGGGTGGC	CCGCTGCCCACCGGATAGC
βIII-tubulin (TUJ1)	qPCR	CATTCTGGTGGACCTGGAAC	CCTCCGTGTAGTGACCCTTG

Table S2. Global *gene expression profiling*

Gene_Name	Ensembl_GeneID	Gene_biotype	Norm_Expr_CTR	Norm_Expr_PD	Fold Change	Log FC	p-Value	FDR	Description
TYW3	ENSG00000162623	protein_coding	821,2679792	2,292202621	0,002791053	-8,48497	5,56E-21	2,2E-16	tRNA-yW synthesizing protein 3 homolog (S. cerevisiae) [Source:HGNC Symbol;Acc:24757]
CRYZ	ENSG00000116791	protein_coding	2082,900517	23,60679145	0,011333614	-6,46325	8,7E-18	1,72E-13	crystallin, zeta (quinone reductase) [Source:HGNC Symbol;Acc:2419]
PABPC4L	ENSG00000254535	protein_coding	0,996597191	181,9964786	182,6178923	7,512684	8E-12	7,92E-08	poly(A) binding protein, cytoplasmic 4-like [Source:HGNC Symbol;Acc:31955]
PCDHA6	ENSG00000081842	protein_coding	10,84352968	161,6453643	14,90708	3,897926	2,63E-07	0,000973	protocadherin alpha 6 [Source:HGNC Symbol;Acc:8672]
ACTG2	ENSG00000163017	protein_coding	1416,568899	148,921766	0,105128502	-3,24977	2,7E-07	0,000973	actin, gamma 2, smooth muscle, enteric [Source:HGNC Symbol;Acc:145]
SST	ENSG00000157005	protein_coding	93,22008073	716,3245039	7,684229602	2,941901	6,73E-06	0,01906	somatostatin [Source:HGNC Symbol;Acc:11329]
CCDC33	ENSG00000140481	protein_coding	3,239947276	60,92945008	18,80569185	4,233097	7,61E-06	0,020116	coiled-coil domain containing 33 [Source:HGNC Symbol;Acc:26552]
ZNF619	ENSG00000177873	protein_coding	3,239947276	60,52504058	18,68087207	4,22349	8,48E-06	0,020995	zinc finger protein 619 [Source:HGNC Symbol;Acc:26910]
CYTL1	ENSG00000170891	protein_coding	10,71845183	108,2772873	10,10195214	3,336562	1,57E-05	0,036643	cytokine-like 1 [Source:HGNC Symbol;Acc:24435]
C5orf17	ENSG00000248874	protein_coding	0	26,42235701	#N/A	#N/A	1,81E-05	0,039672	chromosome 5 open reading frame 17 [Source:HGNC Symbol;Acc:26630]
PPP1R17	ENSG00000106341	protein_coding	30,15813548	276,7568107	9,176854148	3,198	1,9E-05	0,039672	protein phosphatase 1, regulatory subunit 17 [Source:HGNC Symbol;Acc:16973]
ZNF572	ENSG00000180938	protein_coding	19,56476151	0	0	#N/A	7,07E-05	0,126278	zinc finger protein 572 [Source:HGNC Symbol;Acc:26758]
PLN	ENSG00000198523	protein_coding	154,5389873	24,19810477	0,156582525	-2,675	7,31E-05	0,126278	phospholamban [Source:HGNC Symbol;Acc:9080]
CTHRC1	ENSG00000164932	protein_coding	115,6636456	11,77707099	0,101821717	-3,29588	7,33E-05	0,126278	collagen triple helix repeat containing 1 [Source:HGNC Symbol;Acc:18831]
TAGLN	ENSG00000149591	protein_coding	8540,571183	1643,764133	0,192465363	-2,37733	7,89E-05	0,130341	transgelin [Source:HGNC Symbol;Acc:11553]
RPL9	ENSG00000163682	protein_coding	635,4934631	3410,007919	5,365921314	2,423826	9,79E-05	0,149146	ribosomal protein L9 [Source:HGNC Symbol;Acc:10369]
ITGA2B	ENSG00000005961	protein_coding	59,30759693	335,7542113	5,661234457	2,501117	0,000263	0,325869	integrin, alpha 2b (platelet glycoprotein IIb of IIb/IIIa complex, antigen CD41) [Source:HGNC Symbol;Acc:6138]
COMT	ENSG00000093010	protein_coding	85,136315	755,4212636	8,873079174	3,149435	0,000335	0,390612	catechol-O-methyltransferase [Source:HGNC Symbol;Acc:2228]
SLC25A41	ENSG00000181240	protein_coding	12,59059397	79,67148054	6,327857186	2,661717	0,00037	0,418456	solute carrier family 25, member 41 [Source:HGNC Symbol;Acc:28533]
THEM6	ENSG00000130193	protein_coding	49,35571466	249,3454775	5,052008248	2,336857	0,000384	0,422398	thioesterase superfamily member 6 [Source:HGNC Symbol;Acc:29656]
NOTUM	ENSG00000185269	protein_coding	25,92159101	145,678097	5,619951989	2,490558	0,000402	0,431068	notum pectinacetylesterase homolog (Drosophila) [Source:HGNC Symbol;Acc:27106]
ART5	ENSG00000167311	protein_coding	16,69602217	108,3656389	6,490506413	2,698331	0,000426	0,43118	ADP-ribosyltransferase 5 [Source:HGNC Symbol;Acc:24049]
YBX2	ENSG00000006047	protein_coding	50,34023501	259,1464038	5,147898172	2,363984	0,000508	0,47714	Y box binding protein 2 [Source:HGNC Symbol;Acc:17948]
ARL17B	ENSG00000228696	protein_coding	7,228348849	56,13773978	7,766329622	2,957233	0,000518	0,47714	ADP-ribosylation factor-like 17B [Source:HGNC Symbol;Acc:32387]
TMEM132E	ENSG00000181291	protein_coding	72,65067081	336,8825421	4,637019016	2,213198	0,000679	0,61087	transmembrane protein 132E [Source:HGNC Symbol;Acc:26991]
ACTA2	ENSG00000107796	protein_coding	2575,820481	640,9858671	0,248847259	-2,00667	0,00079	0,679576	actin, alpha 2, smooth muscle, aorta [Source:HGNC Symbol;Acc:130]
SPP1	ENSG00000118785	protein_coding	4791,316164	1161,146067	0,242343863	-2,04487	0,000806	0,679576	secreted phosphoprotein 1 [Source:HGNC Symbol;Acc:11255]
TNFRSF1B	ENSG00000028137	protein_coding	28,54017465	138,2934271	4,845570456	2,276667	0,00104	0,840968	tumor necrosis factor receptor superfamily, member 1B [Source:HGNC Symbol;Acc:11917]
TNC	ENSG00000041982	protein_coding	9705,32392	2549,610142	0,26270222	-1,9285	0,00104	0,840968	tenascin C [Source:HGNC Symbol;Acc:5318]
GPR50	ENSG00000102195	protein_coding	190,8158734	43,04378777	0,225577605	-2,1483	0,001153	0,885745	G protein-coupled receptor 50 [Source:HGNC Symbol;Acc:4506]
DCDC2	ENSG00000146038	protein_coding	31,53197903	149,6252861	4,74519173	2,246466	0,001158	0,885745	doublecortin domain containing 2 [Source:HGNC Symbol;Acc:18141]
LY6H	ENSG00000176956	protein_coding	88,2431332	362,066169	4,103052055	2,036697	0,001162	0,885745	lymphocyte antigen 6 complex, locus H [Source:HGNC Symbol;Acc:6728]
PTGR1	ENSG00000106853	protein_coding	1098,098009	283,0131498	0,257730319	-1,95607	0,001217	0,909592	prostaglandin reductase 1 [Source:HGNC Symbol;Acc:18429]
ZP3	ENSG00000188372	protein_coding	43,1219502	5,683780594	0,131807132	-2,9235	0,001263	0,92647	zona pellucida glycoprotein 3 (sperm receptor) [Source:HGNC Symbol;Acc:13189]
CRHR1	ENSG00000120088	protein_coding	8,723244636	55,45297454	6,356920716	2,668328	0,001354	0,957774	corticotropin releasing hormone receptor 1 [Source:HGNC Symbol;Acc:2357]
TMEM121	ENSG00000184986	protein_coding	9,350646696	53,04691878	5,673074869	2,504131	0,001448	1	transmembrane protein 121 [Source:HGNC Symbol;Acc:20511]
CST1	ENSG00000170373	protein_coding	42,37550871	185,1909521	4,370235491	2,127711	0,001622	1	cystatin SN [Source:HGNC Symbol;Acc:2473]
TULP1	ENSG00000112041	protein_coding	10,71643902	69,51714785	6,486963414	2,697543	0,00165	1	tubby like protein 1 [Source:HGNC Symbol;Acc:12423]
LRRC53	ENSG00000162621	protein_coding	2,74164868	27,82758964	10,14994731	3,3434	0,001924	1	leucine rich repeat containing 53 [Source:HGNC Symbol;Acc:25255]
ATP2B2	ENSG00000157087	protein_coding	9,721854634	116,2057214	11,95304042	3,579306	0,001926	1	ATPase, Ca++ transporting, plasma membrane 2 [Source:HGNC Symbol;Acc:815]
LGI4	ENSG00000153902	protein_coding	104,4187159	420,9413234	4,031282321	2,011239	0,001981	1	leucine-rich repeat LGI family, member 4 [Source:HGNC Symbol;Acc:18712]
TMBIM4	ENSG00000155957	protein_coding	73,05609647	264,0944161	3,614953835	1,853977	0,002136	1	transmembrane BAX inhibitor motif containing 4 [Source:HGNC Symbol;Acc:24257]
NTRK1	ENSG00000198400	protein_coding	31,65504407	136,3172823	4,306336836	2,106461	0,002167	1	neurotrophic tyrosine kinase, receptor, type 1 [Source:HGNC Symbol;Acc:8031]
LILRA6	ENSG00000244482	protein_coding	60,82463359	8,774601598	0,144260657	-2,79325	0,002242	1	leukocyte immunoglobulin-like receptor, subfamily A (with TM domain), member 6 [Source:HGNC Symbol;Acc:15495]
AGPAT9	ENSG00000138678	protein_coding	76,88922808	16,83893642	0,219002542	-2,19098	0,002343	1	1-acylglycerol-3-phosphate O-acyltransferase 9 [Source:HGNC Symbol;Acc:28157]
POU4F1	ENSG00000152192	protein_coding	134,0886168	505,7307727	3,771616001	1,915183	0,002347	1	POU class 4 homeobox 1 [Source:HGNC Symbol;Acc:9218]
FOXN4	ENSG00000139445	protein_coding	12,09028257	61,9251729	5,121896246	2,356678	0,002379	1	forkhead box N4 [Source:HGNC Symbol;Acc:21399]
PALMD	ENSG00000099260	protein_coding	106,8012335	25,80044185	0,241574381	-2,04946	0,00238	1	palmdelphin [Source:HGNC Symbol;Acc:15846]
ZFR2	ENSG00000105278	protein_coding	70,41134634	278,9555614	3,96179843	1,986155	0,002385	1	zinc finger RNA binding protein 2 [Source:HGNC Symbol;Acc:29189]
ALOX15B	ENSG00000179593	protein_coding	41,49392533	3,785786858	0,091237135	-3,45424	0,002392	1	arachidonate 15-lipoxygenase, type B [Source:HGNC Symbol;Acc:434]
CRIP3	ENSG00000146215	protein_coding	8,84630968	54,22444512	6,129611905	2,615796	0,002701	1	cysteine-rich protein 3 [Source:HGNC Symbol;Acc:17751]
NEUROD1	ENSG00000162992	protein_coding	30,91061539	255,4473221	8,264064591	3,046852	0,00271	1	neuronal differentiation 1 [Source:HGNC Symbol;Acc:7762]
C8orf31	ENSG00000177335	protein_coding	8,602192399	45,77610203	5,321445965	2,411818	0,002753	1	chromosome 8 open reading frame 31 [Source:HGNC Symbol;Acc:26731]
RGPD2	ENSG00000185304	protein_coding	19,55872308	98,20110564	5,020834193	2,327927	0,002821	1	RANBP2-like and GRIP domain containing 2 [Source:HGNC Symbol;Acc:32415]
NNMT	ENSG00000166741	protein_coding	616,3263598	130,7863498	0,212203077	-2,23648	0,003097	1	nicotinamide N-methyltransferase [Source:HGNC Symbol;Acc:7861]
IAH1	ENSG00000134330	protein_coding	74,30083655	488,0354682	6,568371109	2,715536	0,003128	1	isoamyl acetate-hydrolyzing esterase 1 homolog (S. cerevisiae) [Source:HGNC Symbol;Acc:27696]
CDC20B	ENSG00000164287	protein_coding	30,90256416	135,3589081	4,380183707	2,130991	0,003179	1	cell division cycle 20B [Source:HGNC Symbol;Acc:24222]
ITGA11	ENSG00000137809	protein_coding	339,1878027	68,94788214	0,203273471	-2,29851	0,003397	1	integrin, alpha 11 [Source:HGNC Symbol;Acc:6136]
EBF4	ENSG00000088881	protein_coding	143,074094	488,8918363	3,417053519	1,772753	0,003847	1	early B-cell factor 4 [Source:HGNC Symbol;Acc:29278]
SAMSN1	ENSG00000155307	protein_coding	1,494895787	20,44291976	13,67514708	3,773484	0,003892	1	SAM domain, SH3 domain and nuclear localization signals 1 [Source:HGNC Symbol;Acc:10528]
HLA-DOA	ENSG00000204252	protein_coding	26,42190242	107,2866648	4,060520062	2,021665	0,003947	1	major histocompatibility complex, class II, DO alpha [Source:HGNC Symbol;Acc:4936]
UPK1B	ENSG00000114638	protein_coding	47,85880607	9,267362704	0,193639655	-2,36855	0,004127	1	uroplakin 1B [Source:HGNC Symbol;Acc:12578]
SLFN12	ENSG00000172123	protein_coding	92,09438008	22,72492176	0,246756879	-2,01884	0,004173	1	schlafen family member 12 [Source:HGNC Symbol;Acc:25500]
HLA-DQB1	ENSG00000179344	protein_coding	2,368427935	22,53291763	9,513870908	3,250032	0,004315	1	major histocompatibility complex, class II, DQ beta 1 [Source:HGNC Symbol;Acc:4944]
OSGIN1	ENSG00000140961	protein_coding	113,7814394	26,74351521	0,235042862	-2,089	0,004523	1	oxidative stress induced growth inhibitor 1 [Source:HGNC Symbol;Acc:30093]
GFRA2	ENSG00000168546	protein_coding	96,33897577	352,9636625	3,663768062	1,873328	0,004646	1	GDNF family receptor alpha 2 [Source:HGNC Symbol;Acc:4244]
TLR5	ENSG00000187554	protein_coding	8,848322486	45,57389728	5,150569201	2,364732	0,005252	1	toll-like receptor 5 [Source:HGNC Symbol;Acc:11851]
LDHD	ENSG00000166816	protein_coding	9,344608275	50,54231079	5,408713599	2,435286	0,005267	1	lactate dehydrogenase D [Source:HGNC Symbol;Acc:19708]
COL11A2	ENSG00000204248	protein_coding	461,309202	2821,636411	6,116583841	2,612726	0,005392	1	collagen, type XI, alpha 2 [Source:HGNC Symbol;Acc:2187]
CA7	ENSG00000168748	protein_coding	0,996597191	17,24334592	17,30222207	4,112885	0,005405	1	carbonic anhydrase VII [Source:HGNC Symbol;Acc:1381]
KCTD19	ENSG00000168676	protein_coding	9,469686126	87,67806559	9,258814329	3,210827	0,005415	1	potassium channel tetramerization domain containing 19 [Source:HGNC Symbol;Acc:24753]
RPS28	ENSG00000233927	protein_coding	544,9969158	161,5213106	0,296371054	-1,75452	0,005434	1	ribosomal protein S28 [Source:HGNC Symbol;Acc:10418]

OR2W3	ENSG00000238243	protein_coding	9,594763977	0	0	#N/A	0,005458	1	olfactory receptor, family 2, subfamily W, member 3 [Source:HGNC Symbol;Acc:15021]
KCNH6	ENSG00000173826	protein_coding	3,239947276	25,62883894	7,910264198	2,983726	0,005683	1	potassium voltage-gated channel, subfamily H (eag-related), member 6 [Source:HGNC Symbol;Acc:18862]
KCNK5	ENSG00000164626	protein_coding	169,3306624	581,2018563	3,432348566	1,779196	0,005808	1	potassium channel, subfamily K, member 5 [Source:HGNC Symbol;Acc:6280]
ENDOD1	ENSG00000149218	protein_coding	1614,619444	539,0991733	0,333886214	-1,58257	0,005891	1	endonuclease domain containing 1 [Source:HGNC Symbol;Acc:29129]
ATP2A3	ENSG00000074370	protein_coding	27,41849961	207,6746742	7,574253775	2,921104	0,005996	1	ATPase, Ca++ transporting, ubiquitous [Source:HGNC Symbol;Acc:813]
C20orf195	ENSG00000125531	protein_coding	9,471698933	45,97830678	4,854282965	2,279258	0,006101	1	chromosome 20 open reading frame 195 [Source:HGNC Symbol;Acc:28764]
CCDC88B	ENSG00000168071	protein_coding	122,8738792	405,1083104	3,296944095	1,721129	0,006145	1	coiled-coil domain containing 88B [Source:HGNC Symbol;Acc:26757]
FGF19	ENSG00000162344	protein_coding	30,91061539	110,8906482	3,587461678	1,842963	0,006426	1	fibroblast growth factor 19 [Source:HGNC Symbol;Acc:3675]
ADM	ENSG00000148926	protein_coding	531,2058635	160,2468784	0,301666245	-1,72897	0,006712	1	adrenomedullin [Source:HGNC Symbol;Acc:259]
HRC	ENSG00000130528	protein_coding	32,89777135	122,0662052	3,710470351	1,891602	0,006751	1	histidine rich calcium binding protein [Source:HGNC Symbol;Acc:5178]
DNAH2	ENSG00000183914	protein_coding	104,4348183	347,3290775	3,325797689	1,7337	0,006786	1	dynein, axonemal, heavy chain 2 [Source:HGNC Symbol;Acc:2948]
MS4A8	ENSG00000166959	protein_coding	5,981595956	34,79764942	5,817452345	2,540387	0,006891	1	membrane-spanning 4-domains, subfamily A, member 8 [Source:HGNC Symbol;Acc:13380]
PRDM12	ENSG00000130711	protein_coding	58,69227171	363,4697553	6,192804345	2,630593	0,006995	1	PR domain containing 12 [Source:HGNC Symbol;Acc:13997]
PDIA2	ENSG00000185615	protein_coding	9,346621082	50,8685693	5,442455498	2,444258	0,007146	1	protein disulfide isomerase family A, member 2 [Source:HGNC Symbol;Acc:14180]
MYOCD	ENSG00000141052	protein_coding	109,554959	30,29139517	0,276494971	-1,85467	0,007181	1	myocardin [Source:HGNC Symbol;Acc:16067]
BEST3	ENSG00000127325	protein_coding	21,18473514	2,790064033	0,131701625	-2,92465	0,007235	1	bestrophin 3 [Source:HGNC Symbol;Acc:17105]
MLXIPL	ENSG00000009950	protein_coding	19,31661861	78,94081254	4,086678633	2,030929	0,007332	1	MLX interacting protein-like [Source:HGNC Symbol;Acc:12744]
NELL1	ENSG00000165973	protein_coding	742,8254839	243,1536349	0,327336151	-1,61116	0,007379	1	NEL-like 1 (chicken) [Source:HGNC Symbol;Acc:7750]
ABCC6	ENSG00000091262	protein_coding	68,78533428	241,3966422	3,50942021	1,811233	0,007689	1	ATP-binding cassette, sub-family C (CFTR/MRP), member 6 [Source:HGNC Symbol;Acc:57]
CALB1	ENSG00000104327	protein_coding	314,4603478	97,56388955	0,310258162	-1,68846	0,007929	1	calbindin 1, 28kDa [Source:HGNC Symbol;Acc:1434]
RASL12	ENSG00000103710	protein_coding	23,42204681	96,40166413	4,115851399	2,041191	0,007972	1	RAS-like, family 12 [Source:HGNC Symbol;Acc:30289]
GLIPR1	ENSG00000139278	protein_coding	889,1996256	153,5928765	0,172731603	-2,5334	0,008094	1	GLI pathogenesis-related 1 [Source:HGNC Symbol;Acc:17001]
F2RL2	ENSG00000164220	protein_coding	315,0716474	99,75243965	0,3166024	-1,65926	0,008133	1	coagulation factor II (thrombin) receptor-like 2 [Source:HGNC Symbol;Acc:3539]
RAB26	ENSG00000167964	protein_coding	39,74887384	141,7156069	3,565273508	1,834013	0,008182	1	RAB26, member RAS oncogene family [Source:HGNC Symbol;Acc:14259]
SYNM	ENSG00000182253	protein_coding	265,7219712	79,66127993	0,299791845	-1,73797	0,00832	1	synemin, intermediate filament protein [Source:HGNC Symbol;Acc:24466]
HLA-DPB1	ENSG00000223865	protein_coding	69,54586542	222,0565518	3,192951161	1,67489	0,008426	1	major histocompatibility complex, class II, DP beta 1 [Source:HGNC Symbol;Acc:4940]
PXMP4	ENSG00000101417	protein_coding	41,00367796	9,459366839	0,230695569	-2,11594	0,008588	1	peroxisomal membrane protein 4, 24kDa [Source:HGNC Symbol;Acc:15920]
HEYL	ENSG00000163909	protein_coding	135,815553	440,3528182	3,242285646	1,697011	0,009113	1	hairy/enhancer-of-split related with YRPW motif-like [Source:HGNC Symbol;Acc:4882]
CALN1	ENSG00000183166	protein_coding	11,21876323	62,7390922	5,592335887	2,483451	0,00912	1	calneuron 1 [Source:HGNC Symbol;Acc:13248]
CBFA2T3	ENSG00000129993	protein_coding	30,16014829	105,0046628	3,481569846	1,799738	0,009302	1	core-binding factor, runt domain, alpha subunit 2; translocated to, 3 [Source:HGNC Symbol;Acc:1537]
TCHH	ENSG00000159450	protein_coding	0	19,2551928	#N/A	#N/A	0,009456	1	trichohyalin [Source:HGNC Symbol;Acc:11791]
TNFRSF12A	ENSG00000006327	protein_coding	1417,776841	377,8345244	0,266497881	-1,9078	0,00952	1	tumor necrosis factor receptor superfamily, member 12A [Source:HGNC Symbol;Acc:18152]
NR4A1	ENSG00000123358	protein_coding	312,965452	893,3119276	2,854346772	1,513161	0,010215	1	nuclear receptor subfamily 4, group A, member 1 [Source:HGNC Symbol;Acc:7980]
SMIM17	ENSG00000268182	protein_coding	4,982985957	38,00232356	7,626415946	2,931005	0,01024	1	small integral membrane protein 17 [Source:HGNC Symbol;Acc:27114]
VWDE	ENSG00000146530	protein_coding	36,75908227	130,001386	3,536578662	1,822354	0,010341	1	von Willebrand factor D and EGF domains [Source:HGNC Symbol;Acc:21897]
TNFAIP8L3	ENSG00000183578	protein_coding	23,0588901	3,386477667	0,146862128	-2,76747	0,010462	1	tumor necrosis factor, alpha-induced protein 8-like 3 [Source:HGNC Symbol;Acc:20620]
GABRQ	ENSG00000147402	protein_coding	24,29960457	90,56158154	3,726874702	1,897966	0,010639	1	gamma-aminobutyric acid (GABA) A receptor, theta [Source:HGNC Symbol;Acc:14454]
CAV1	ENSG00000105974	protein_coding	983,4588563	343,5618844	0,349340374	-1,51729	0,010657	1	caveolin 1, caveolae protein, 22kDa [Source:HGNC Symbol;Acc:1527]
FLT1	ENSG00000102755	protein_coding	1157,207783	426,2697289	0,368360579	-1,44081	0,010694	1	fms-related tyrosine kinase 1 [Source:HGNC Symbol;Acc:3763]
USH1C	ENSG00000006611	protein_coding	17,32141142	67,50530097	3,897217111	1,962444	0,010726	1	Usher syndrome 1C (autosomal recessive, severe) [Source:HGNC Symbol;Acc:12597]
PDYN	ENSG00000101327	protein_coding	19,93798225	75,19072783	3,771230553	1,915035	0,010761	1	prodynorphin [Source:HGNC Symbol;Acc:8820]
C17orf102	ENSG00000197322	protein_coding	1,619973638	16,6571329	10,28234812	3,362098	0,0109	1	chromosome 17 open reading frame 102 [Source:HGNC Symbol;Acc:34412]
LAMC2	ENSG00000058085	protein_coding	598,6052774	207,8444696	0,347214563	-1,5261	0,011049	1	laminin, gamma 2 [Source:HGNC Symbol;Acc:6493]
CD164L2	ENSG00000174950	protein_coding	1,246752893	14,75403886	11,83397202	3,564862	0,011082	1	CD164 sialomucin-like 2 [Source:HGNC Symbol;Acc:32043]
COLCA2	ENSG00000214290	protein_coding	6,730050253	33,30916549	4,949318985	2,30723	0,011083	1	colorectal cancer associated 2 [Source:HGNC Symbol;Acc:26978]
GBP1	ENSG00000117228	protein_coding	147,0403547	42,04806494	0,285962755	-1,8061	0,011256	1	guanylate binding protein 1, interferon-inducible [Source:HGNC Symbol;Acc:4182]
CDK18	ENSG00000117266	protein_coding	299,0415545	926,085722	3,096846268	1,6308	0,011759	1	cyclin-dependent kinase 18 [Source:HGNC Symbol;Acc:8751]
CADM2	ENSG00000175161	protein_coding	83,61726553	257,4761163	3,079221913	1,622566	0,012165	1	cell adhesion molecule 2 [Source:HGNC Symbol;Acc:29849]
FGF1	ENSG00000113578	protein_coding	45,6194816	6,881708168	0,150850205	-2,72881	0,012741	1	fibroblast growth factor 1 (acidic) [Source:HGNC Symbol;Acc:3665]
INHBA	ENSG00000122641	protein_coding	275,6899559	95,44328984	0,346197922	-1,53033	0,013133	1	inhibin, beta A [Source:HGNC Symbol;Acc:6066]
CDHR2	ENSG00000074276	protein_coding	23,55517588	79,48967702	3,374616153	1,754723	0,013319	1	cadherin-related family member 2 [Source:HGNC Symbol;Acc:18231]
ZNF280D	ENSG00000137871	protein_coding	220,1369911	1054,814644	4,791628336	2,260516	0,013341	1	zinc finger protein 280D [Source:HGNC Symbol;Acc:25953]
NBPF14	ENSG00000122497	protein_coding	221,3391784	628,9914516	2,841753801	1,506782	0,013393	1	neuroblastoma breakpoint family, member 14 [Source:HGNC Symbol;Acc:25232]
MAP3K7CL	ENSG00000156265	protein_coding	82,8587472	5,180818875	0,062525914	-3,9994	0,013714	1	MAP3K7 C-terminal like [Source:HGNC Symbol;Acc:16457]
POLR2J3	ENSG00000168255	protein_coding	102,4375984	31,50462367	0,307549417	-1,70111	0,013778	1	polymerase (RNA) II (DNA directed) polypeptide J3 [Source:HGNC Symbol;Acc:33853]
ADAM11	ENSG00000073670	protein_coding	196,3052092	724,6167061	3,691275993	1,88412	0,013908	1	ADAM metallopeptidase domain 11 [Source:HGNC Symbol;Acc:189]
SGMS2	ENSG00000164023	protein_coding	262,8149885	93,48754634	0,35571619	-1,4912	0,014495	1	sphingomyelin synthase 2 [Source:HGNC Symbol;Acc:28395]
PTGS1	ENSG00000095303	protein_coding	39,8759645	132,0760829	3,312172748	1,727778	0,014517	1	prostaglandin-endoperoxide synthase 1 (prostaglandin G/H synthase and cyclooxygenase) [Source:HGNC Symbol;Acc:9604]
NQO1	ENSG00000181019	protein_coding	3876,147234	1076,193891	0,277645256	-1,84869	0,01461	1	NAD(P)H dehydrogenase, quinone 1 [Source:HGNC Symbol;Acc:2874]
ADAMTS4	ENSG00000158859	protein_coding	70,41134634	234,0868306	3,324561207	1,733164	0,014936	1	ADAM metallopeptidase with thrombospondin type 1 motif, 4 [Source:HGNC Symbol;Acc:220]
SYT7	ENSG00000011347	protein_coding	153,4173123	437,9637097	2,854721565	1,51335	0,015105	1	synaptotagmin VII [Source:HGNC Symbol;Acc:11514]
KIAA1456	ENSG00000250305	protein_coding	288,9827096	820,3828005	2,838864656	1,505314	0,015214	1	KIAA1456 [Source:HGNC Symbol;Acc:26725]
WDR86	ENSG00000187260	protein_coding	172,6936747	529,8913678	3,068388976	1,617481	0,015257	1	WD repeat domain 86 [Source:HGNC Symbol;Acc:28020]
CD8B	ENSG00000172116	protein_coding	2,243350084	17,84485986	7,954558668	2,991782	0,015555	1	CD8b molecule [Source:HGNC Symbol;Acc:1707]
RNF144B	ENSG00000137393	protein_coding	68,42821599	15,85341421	0,23167949	-2,1098	0,015585	1	ring finger protein 144B [Source:HGNC Symbol;Acc:21578]
KCNQ1	ENSG00000053918	protein_coding	12,83269845	52,74616181	4,110293874	2,039242	0,015587	1	potassium voltage-gated channel, KQT-like subfamily, member 1 [Source:HGNC Symbol;Acc:6294]
CAMK1G	ENSG00000008118	protein_coding	72,39045107	232,248233	3,208271665	1,681796	0,015597	1	calcium/calmodulin-dependent protein kinase IG [Source:HGNC Symbol;Acc:14585]
ERAP2	ENSG00000164308	protein_coding	67,29043849	21,12258469	0,313901725	-1,67162	0,015796	1	endoplasmic reticulum aminopeptidase 2 [Source:HGNC Symbol;Acc:29499]
SELV	ENSG00000186838	protein_coding	12,08826976	46,47616819	3,844732878	1,942883	0,016689	1	Selenoprotein V [Source:UniProtKB/Swiss-Prot;Acc:P59797]
ATG16L2	ENSG00000168010	protein_coding	269,3109855	745,4214254	2,767883471	1,468783	0,016745	1	autophagy related 16-like 2 (S. cerevisiae) [Source:HGNC Symbol;Acc:25464]
EYA2	ENSG00000064655	protein_coding	79,77406988	263,5065567	3,303160502	1,723847	0,016748	1	eyes absent homolog 2 (Drosophila) [Source:HGNC Symbol;Acc:3520]
OAS3	ENSG00000111331	protein_coding	8,350023891	35,99557699	4,31083521	2,107967	0,016881	1	2'-5'-oligoadenylate synthetase 3, 100kDa [Source:HGNC Symbol;Acc:8088]
HYAL1	ENSG00000114378	protein_coding	34,89096574	116,606677	3,342030653	1,740725	0,017292	1	hyaluronoglucosaminidase 1 [Source:HGNC Symbol;Acc:5320]
NEUROG1	ENSG00000181965	protein_coding	27,91881101	225,2493789	8,068014745	3,012214	0,017308	1	neurogenin 1 [Source:HGNC Symbol;Acc:7764]
IGLON5	ENSG00000142549	protein_coding	531,2967236	1920,035735	3,613867072	1,853543	0,017397	1	IgLON family member 5 [Source:HGNC Symbol;Acc:34550]
ADAMTS16	ENSG00000145536	protein_coding	156,4091166	50,66126424	0,323902246	-1,62637	0,017423	1	ADAM metallopeptidase with thrombospondin type 1 motif, 16 [Source:HGNC Symbol;Acc:17108]
GCLM	ENSG00000023909	protein_coding	1093,462077	413,1453362	0,377832341	-1,40418	0,017963	1	glutamate-cysteine ligase, modifier subunit [Source:HGNC Symbol;Acc:4312]
PDLIM3	ENSG00000154553	protein_coding	380,4010964	117,1198393	0,307885126	-1,69954	0,017972	1	PDZ and LIM domain 3 [Source:HGNC Symbol;Acc:20767]
TLX2	ENSG00000115297	protein_coding	14,58378835	51,83369028	3,554199295	1,829525	0,018218	1	T-cell leukemia homeobox 2 [Source:HGNC Symbol;Acc:5057]
GRIK4	ENSG00000149403	protein_coding	26,9181882	87,34160648	3,244705989	1,698088	0,018436	1	glutamate receptor, ionotropic, kainate 4 [Source:HGNC Symbol;Acc:4582]

ZNF680	ENSG00000173041	protein_coding	159,4250747	414,5064736	2,600008025	1,378516	0,018528	1	zinc finger protein 680 [Source:HGNC Symbol;Acc:26897]
ME1	ENSG00000065833	protein_coding	862,4513646	337,9393074	0,391835785	-1,35168	0,01853	1	malic enzyme 1, NADP(+)-dependent, cytosolic [Source:HGNC Symbol;Acc:6983]
AP003068.23	ENSG00000254614	protein_coding	19,69185216	3,090821003	0,156959385	-2,67154	0,018661	1	Uncharacterized protein [Source:UniProtKB/TrEMBL;Acc:E9PK70]
CDH23	ENSG00000107736	protein_coding	73,90144932	208,6346948	2,82314754	1,497305	0,01908	1	cadherin-related 23 [Source:HGNC Symbol;Acc:13733]
LMCD1	ENSG00000071282	protein_coding	2481,36975	999,9541462	0,402984741	-1,3112	0,019274	1	LIM and cysteine-rich domains 1 [Source:HGNC Symbol;Acc:6633]
SERPINB8	ENSG00000166401	protein_coding	203,904766	69,68365045	0,34174606	-1,549	0,019458	1	serpin peptidase inhibitor, clade B (ovalbumin), member 8 [Source:HGNC Symbol;Acc:8952]
KLHL40	ENSG00000157119	protein_coding	0	23,6475939	#N/A	#N/A	0,019486	1	kelch-like family member 40 [Source:HGNC Symbol;Acc:30372]
TXNRD2	ENSG00000184470	protein_coding	46,73511822	144,0231104	3,081689229	1,623721	0,019533	1	thioredoxin reductase 2 [Source:HGNC Symbol;Acc:18155]
GTF2H2	ENSG00000145736	protein_coding	118,5122569	334,78399	2,824889162	1,498194	0,019562	1	general transcription factor IIH, polypeptide 2, 44kDa [Source:HGNC Symbol;Acc:4656]
SLFN13	ENSG00000154760	protein_coding	89,97812065	255,6154711	2,840862526	1,506329	0,019631	1	schlafen family member 13 [Source:HGNC Symbol;Acc:26481]
PCSK1N	ENSG00000102109	protein_coding	187,711068	38,30818084	0,204080565	-2,29279	0,019637	1	proprotein convertase subtilisin/kexin type 1 inhibitor [Source:HGNC Symbol;Acc:17301]
THSD7B	ENSG00000144229	protein_coding	65,4343988	180,5998001	2,760013134	1,464675	0,019675	1	thrombospondin, type I, domain containing 7B [Source:HGNC Symbol;Acc:29348]
RGS11	ENSG00000076344	protein_coding	37,00923797	116,1547183	3,138533099	1,65009	0,019693	1	regulator of G-protein signaling 11 [Source:HGNC Symbol;Acc:9993]
GDF3	ENSG00000184344	protein_coding	9,846932485	0,497861413	0,050560051	-4,30586	0,020209	1	growth differentiation factor 3 [Source:HGNC Symbol;Acc:4218]
ISL1	ENSG00000016082	protein_coding	90,83756315	261,6612124	2,88053976	1,526339	0,020482	1	ISL LIM homeobox 1 [Source:HGNC Symbol;Acc:6132]
UCN	ENSG00000163794	protein_coding	7,976803146	33,29896488	4,174474945	2,061595	0,020705	1	urocortin [Source:HGNC Symbol;Acc:12516]
CERKL	ENSG00000188452	protein_coding	105,9357526	314,6758829	2,970440813	1,570677	0,020801	1	ceramide kinase-like [Source:HGNC Symbol;Acc:21699]
N4BP2L1	ENSG00000139597	protein_coding	88,72734214	245,1485345	2,762942387	1,466205	0,021381	1	NEDD4 binding protein 2-like 1 [Source:HGNC Symbol;Acc:25037]
ABHD12B	ENSG00000131969	protein_coding	6,356829508	28,118146	4,423297175	2,145122	0,021435	1	abhydrolase domain containing 12B [Source:HGNC Symbol;Acc:19837]
ITGA4	ENSG00000115232	protein_coding	224,6881011	711,1438461	3,165026731	1,662218	0,021486	1	integrin, alpha 4 (antigen CD49D, alpha 4 subunit of VLA-4 receptor) [Source:HGNC Symbol;Acc:6140]
MAP7D3	ENSG00000129680	protein_coding	598,1676468	225,7437864	0,37739217	-1,40586	0,021542	1	MAP7 domain containing 3 [Source:HGNC Symbol;Acc:25742]
NPPA	ENSG00000175206	protein_coding	40,25924928	8,365091793	0,20778062	-2,26687	0,021751	1	natriuretic peptide A [Source:HGNC Symbol;Acc:7939]
AIFM3	ENSG00000183773	protein_coding	13,83332125	48,66981859	3,518303212	1,81488	0,021761	1	apoptosis-inducing factor, mitochondrion-associated, 3 [Source:HGNC Symbol;Acc:26398]
SYCP2L	ENSG00000153157	protein_coding	94,57782183	269,6065938	2,8506323	1,511282	0,021812	1	synaptonemal complex protein 2-like [Source:HGNC Symbol;Acc:21537]
KCNC1	ENSG00000129159	protein_coding	85,86061561	238,6457343	2,779455197	1,474802	0,021827	1	potassium voltage-gated channel, Shaw-related subfamily, member 1 [Source:HGNC Symbol;Acc:6233]
PCSK9	ENSG00000169174	protein_coding	188,3870613	543,3115784	2,884017483	1,52808	0,022034	1	proprotein convertase subtilisin/kexin type 9 [Source:HGNC Symbol;Acc:20001]
JAKMIP1	ENSG00000152969	protein_coding	4,734843063	24,63311611	5,20252008	2,379211	0,022153	1	janus kinase and microtubule interacting protein 1 [Source:HGNC Symbol;Acc:26460]
ERBB3	ENSG00000065361	protein_coding	1064,614821	2652,641918	2,491644739	1,317098	0,022413	1	v-erb-b2 avian erythroblastic leukemia viral oncogene homolog 3 [Source:HGNC Symbol;Acc:3431]
IL1RAP	ENSG00000196083	protein_coding	279,5210747	105,9544829	0,379057225	-1,39951	0,022428	1	interleukin 1 receptor accessory protein [Source:HGNC Symbol;Acc:5995]
ERVV-1	ENSG00000269526	protein_coding	13,95638629	1,695788987	0,121506309	-3,0409	0,022967	1	endogenous retrovirus group V, member 1 [Source:HGNC Symbol;Acc:26501]
NTN5	ENSG00000142233	protein_coding	19,06847572	103,9409896	5,450933318	2,446503	0,023028	1	netrin 5 [Source:HGNC Symbol;Acc:25208]
ALPL	ENSG00000162551	protein_coding	391,0692281	1025,557806	2,622445675	1,390913	0,023643	1	alkaline phosphatase, liver/bone/kidney [Source:HGNC Symbol;Acc:438]
EDN1	ENSG00000078401	protein_coding	360,4976157	118,5675208	0,328899598	-1,60428	0,023772	1	endothelin 1 [Source:HGNC Symbol;Acc:3176]
ZNF717	ENSG00000227124	protein_coding	95,5985727	23,30093417	0,24373726	-2,0366	0,02428	1	zinc finger protein 717 [Source:HGNC Symbol;Acc:29448]
DCST2	ENSG00000163354	protein_coding	15,45329489	60,14613261	3,892123528	1,960557	0,024768	1	DC-STAMP domain containing 2 [Source:HGNC Symbol;Acc:26562]
RNASE1	ENSG00000129538	protein_coding	32,76866789	165,6792587	5,056026667	2,338004	0,024897	1	ribonuclease, RNase A family, 1 (pancreatic) [Source:HGNC Symbol;Acc:10044]
RNF157	ENSG00000141576	protein_coding	375,2467381	952,0630282	2,537165368	1,343218	0,024982	1	ring finger protein 157 [Source:HGNC Symbol;Acc:29402]
ATP8B3	ENSG00000130270	protein_coding	56,19675312	170,3469152	3,031259028	1,599917	0,025097	1	ATPase, aminophospholipid transporter, class I, type 8B, member 3 [Source:HGNC Symbol;Acc:13535]
DTX1	ENSG00000135144	protein_coding	50,96964988	159,6845205	3,13293344	1,647514	0,02534	1	deltex homolog 1 (Drosophila) [Source:HGNC Symbol;Acc:3060]
NSMCE1	ENSG00000169189	protein_coding	194,5380169	504,2286343	2,591928521	1,374026	0,025952	1	non-SMC element 1 homolog (S. cerevisiae) [Source:HGNC Symbol;Acc:29897]
C1orf111	ENSG00000171722	protein_coding	3,988401573	21,24663845	5,327106125	2,413352	0,025977	1	chromosome 1 open reading frame 111 [Source:HGNC Symbol;Acc:27648]
KB-1507C5.2	ENSG00000253320	protein_coding	25,42127961	79,46417548	3,125892036	1,644268	0,026067	1	HCG15011, isoform CRA_a; Protein LOC100996457 [Source:UniProtKB/TrEMBL;Acc:G3V139]
LAPTM5	ENSG00000162511	protein_coding	20,68241093	76,74206183	3,710498843	1,891613	0,026363	1	lysosomal protein transmembrane 5 [Source:HGNC Symbol;Acc:29612]
SOX8	ENSG00000005513	protein_coding	152,5377417	397,854441	2,608236077	1,383074	0,026365	1	SRY (sex determining region Y)-box 8 [Source:HGNC Symbol;Acc:11203]
MPPED1	ENSG00000186732	protein_coding	8,350023891	33,29896488	3,987888575	1,995625	0,026384	1	metallophosphoesterase domain containing 1 [Source:HGNC Symbol;Acc:1306]
FAM129A	ENSG00000135842	protein_coding	625,8342893	250,6011549	0,400427332	-1,32039	0,026612	1	family with sequence similarity 129, member A [Source:HGNC Symbol;Acc:16784]
IL31RA	ENSG00000164509	protein_coding	29,16153829	7,571573717	0,259642466	-1,9454	0,02671	1	interleukin 31 receptor A [Source:HGNC Symbol;Acc:18969]
CDKN1A	ENSG00000124762	protein_coding	4115,037852	1587,222306	0,385712687	-1,3744	0,026911	1	cyclin-dependent kinase inhibitor 1A (p21, Cip1) [Source:HGNC Symbol;Acc:1784]
GBP4	ENSG00000162654	protein_coding	124,3728006	48,40641016	0,38920415	-1,3614	0,027054	1	guanylate binding protein 4 [Source:HGNC Symbol;Acc:20480]
AP001055.1	ENSG00000267913	protein_coding	13,83533406	44,98258395	3,251282822	1,701009	0,027056	1	
SLC25A27	ENSG00000153291	protein_coding	256,7344812	636,612382	2,479652827	1,310138	0,027246	1	solute carrier family 25, member 27 [Source:HGNC Symbol;Acc:21065]
IL10RA	ENSG00000110324	protein_coding	4,73685587	22,4292651	4,735053317	2,243381	0,027322	1	interleukin 10 receptor, alpha [Source:HGNC Symbol;Acc:5964]
GLYATL2	ENSG00000156689	protein_coding	22,05625448	69,11273835	3,13347574	1,647764	0,027467	1	glycine-N-acyltransferase-like 2 [Source:HGNC Symbol;Acc:24178]
IGF1	ENSG00000017427	protein_coding	40,6264316	12,3530834	0,304065184	-1,71755	0,027577	1	insulin-like growth factor 1 (somatomedin C) [Source:HGNC Symbol;Acc:5464]
TIE1	ENSG00000066056	protein_coding	76,87715124	220,014103	2,861891986	1,516969	0,027957	1	tyrosine kinase with immunoglobulin-like and EGF-like domains 1 [Source:HGNC Symbol;Acc:11809]
DOC2A	ENSG00000149927	protein_coding	27,04326605	80,66720337	2,98289427	1,576713	0,028019	1	double C2-like domains, alpha [Source:HGNC Symbol;Acc:2985]
KCNE4	ENSG00000152049	protein_coding	307,3772049	50,70371309	0,164955996	-2,59985	0,028434	1	potassium voltage-gated channel, Isk-related family, member 4 [Source:HGNC Symbol;Acc:6244]
SLC6A3	ENSG00000142319	protein_coding	5,233141659	67,14169392	12,83009295	3,68146	0,02847	1	solute carrier family 6 (neurotransmitter transporter), member 3 [Source:HGNC Symbol;Acc:11049]
AP1S3	ENSG00000152056	protein_coding	153,3971842	60,58789065	0,394973943	-1,34017	0,028503	1	adaptor-related protein complex 1, sigma 3 subunit [Source:HGNC Symbol;Acc:18971]
COLCA1	ENSG00000196167	protein_coding	4,609765212	23,53374076	5,105192928	2,351965	0,028579	1	colorectal cancer associated 1 [Source:HGNC Symbol;Acc:33789]
CHRNB4	ENSG00000117971	protein_coding	41,37891152	111,8965716	2,704193211	1,435198	0,02863	1	cholinergic receptor, nicotinic, beta 4 (neuronal) [Source:HGNC Symbol;Acc:1964]
FLRT2	ENSG00000185070	protein_coding	4883,326574	2112,195619	0,432532125	-1,20912	0,028656	1	fibronectin leucine rich transmembrane protein 2 [Source:HGNC Symbol;Acc:3761]
CGREF1	ENSG00000138028	protein_coding	14,95298349	50,36560758	3,368264776	1,752006	0,029007	1	cell growth regulator with EF-hand domain 1 [Source:HGNC Symbol;Acc:16962]
FBN3	ENSG00000142449	protein_coding	743,8666467	1997,380313	2,685132237	1,424993	0,029295	1	fibrillin 3 [Source:HGNC Symbol;Acc:18794]
C1orf173	ENSG00000178965	protein_coding	4,859920914	23,62719267	4,861641392	2,281443	0,029702	1	chromosome 1 open reading frame 173 [Source:HGNC Symbol;Acc:25346]
SYNGR3	ENSG00000127561	protein_coding	25,17716233	121,2981887	4,817786337	2,26837	0,030259	1	synaptogyrin 3 [Source:HGNC Symbol;Acc:11501]
OGN	ENSG00000106809	protein_coding	33,77532911	9,267362704	0,274382603	-1,86574	0,030626	1	osteoglycin [Source:HGNC Symbol;Acc:8126]
HHATL	ENSG00000010282	protein_coding	0,623376446	9,474667759	15,19895051	3,9259	0,030629	1	hedgehog acyltransferase-like [Source:HGNC Symbol;Acc:13242]
AC016757.3	ENSG00000186235	protein_coding	9,221543231	34,60564528	3,752695662	1,907927	0,030669	1	Protein LOC151174 [Source:UniProtKB/TrEMBL;Acc:E7EUL1]
CTD-3088G3.8	ENSG00000188897	protein_coding	46,85818326	183,0364577	3,906179135	1,965758	0,030964	1	Protein LOC388210 [Source:UniProtKB/TrEMBL;Acc:I3L099]
ISLR2	ENSG00000167178	protein_coding	16,95221629	75,21112905	4,43665464	2,149472	0,031208	1	immunoglobulin superfamily containing leucine-rich repeat 2 [Source:HGNC Symbol;Acc:29286]
NXPH3	ENSG00000182575	protein_coding	22,05424168	71,07358216	3,222671774	1,688257	0,031451	1	neurexophilin 3 [Source:HGNC Symbol;Acc:8077]
CES1	ENSG00000198848	protein_coding	9,221543231	34,99475386	3,794891265	1,924059	0,031623	1	carboxylesterase 1 [Source:HGNC Symbol;Acc:1863]
KCNAB3	ENSG00000170049	protein_coding	113,7874779	281,8441776	2,476934922	1,308556	0,031732	1	potassium voltage-gated channel, shaker-related subfamily, beta member 3 [Source:HGNC Symbol;Acc:6230]
FOXD3	ENSG00000187140	protein_coding	45,98263831	203,9874395	4,436183895	2,149319	0,031795	1	forkhead box D3 [Source:HGNC Symbol;Acc:3804]
LOXL3	ENSG00000115318	protein_coding	356,0974662	938,4271195	2,635309735	1,397973	0,031877	1	lysyl oxidase-like 3 [Source:HGNC Symbol;Acc:13869]
FLRT1	ENSG00000126500	protein_coding	39,74887384	112,9908466	2,842617557	1,50722	0,032093	1	fibronectin leucine rich transmembrane protein 1 [Source:HGNC Symbol;Acc:3760]
ADSSL1	ENSG00000185100	protein_coding	108,0439608	276,3371002	2,557635784	1,354811	0,032337	1	adenylosuccinate synthase like 1 [Source:HGNC Symbol;Acc:20093]
GBP2	ENSG00000162645	protein_coding	168,8766456	67,14334032	0,397588074	-1,33065	0,032367	1	guanylate binding protein 2, interferon-inducible [Source:HGNC Symbol;Acc:4183]
RARRES2	ENSG00000106538	protein_coding	184,8242134	450,2777982	2,436248962	1,284662	0,032475	1	retinoic acid receptor responder (tazarotene induced) 2 [Source:HGNC Symbol;Acc:9868]

GRTP1	ENSG00000139835	protein_coding	104,3278558	256,8117523	2,46158373	1,299587	0,032963	1	growth hormone regulated TBC protein 1 [Source:HGNC Symbol;Acc:20310]
YPEL1	ENSG00000100027	protein_coding	303,5987029	725,2402677	2,388812142	1,256293	0,033125	1	yippee-like 1 (Drosophila) [Source:HGNC Symbol;Acc:12845]
SPINK5	ENSG00000133710	protein_coding	117,7960075	8,572396849	0,072773238	-3,78045	0,033189	1	serine peptidase inhibitor, Kazal type 5 [Source:HGNC Symbol;Acc:15464]
CCDC163P	ENSG00000236624	protein_coding	181,3320977	68,50102379	0,377765573	-1,40444	0,033444	1	coiled-coil domain containing 163, pseudogene [Source:HGNC Symbol;Acc:27003]
MAATS1	ENSG00000183833	protein_coding	59,68685609	484,440039	8,116360465	3,020833	0,033476	1	MYCBP-associated, testis expressed 1 [Source:HGNC Symbol;Acc:24010]
NEUROG2	ENSG00000178403	protein_coding	60,31627096	160,2299311	2,656495976	1,409525	0,033535	1	neurogenin 2 [Source:HGNC Symbol;Acc:13805]
ZNF493	ENSG00000196268	protein_coding	143,7923562	374,4396537	2,604030309	1,380746	0,033714	1	zinc finger protein 493 [Source:HGNC Symbol;Acc:23708]
TEX15	ENSG00000133863	protein_coding	82,86478562	224,6817596	2,711426306	1,439052	0,033726	1	testis expressed 15 [Source:HGNC Symbol;Acc:11738]
PLCG2	ENSG00000197943	protein_coding	60,56642666	160,6190396	2,651948423	1,407053	0,033818	1	phospholipase C, gamma 2 (phosphatidylinositol-specific) [Source:HGNC Symbol;Acc:9066]
MYOM2	ENSG00000036448	protein_coding	122,7608782	48,41151047	0,394356176	-1,34243	0,034178	1	myomesin 2 [Source:HGNC Symbol;Acc:7614]
CHST6	ENSG00000183196	protein_coding	32,64761565	94,61752353	2,898144984	1,53513	0,03427	1	carbohydrate (N-acetylglucosamine 6-O) sulfotransferase 6 [Source:HGNC Symbol;Acc:6938]
ZNF229	ENSG00000167383	protein_coding	0	61,05515023	#N/A	#N/A	0,034519	1	zinc finger protein 229 [Source:HGNC Symbol;Acc:13022]
TUSC1	ENSG00000198680	protein_coding	167,5148789	38,33368237	0,228837478	-2,1276	0,034734	1	tumor suppressor candidate 1 [Source:HGNC Symbol;Acc:31010]
CLEC18C	ENSG00000157335	protein_coding	9,348633889	32,30834236	3,455942627	1,789079	0,035128	1	C-type lectin domain family 18, member C [Source:HGNC Symbol;Acc:28538]
EFHB	ENSG00000163576	protein_coding	13,7082434	54,46235202	3,972963598	1,990216	0,035363	1	EF-hand domain family, member B [Source:HGNC Symbol;Acc:26330]
NR2E1	ENSG00000112333	protein_coding	19,57281273	112,0257257	5,723537398	2,516907	0,035497	1	nuclear receptor subfamily 2, group E, member 1 [Source:HGNC Symbol;Acc:7973]
ITGAL	ENSG00000005844	protein_coding	1,619973638	13,45245875	8,304122017	3,053828	0,035659	1	integrin, alpha L (antigen CD11A (p180), lymphocyte function-associated antigen 1; alpha polypeptide) [Source:HGNC Symbol;Acc:6148]
COL9A2	ENSG00000049089	protein_coding	265,2377622	612,7999319	2,310379664	1,20813	0,0357	1	collagen, type IX, alpha 2 [Source:HGNC Symbol;Acc:2218]
ZNF354C	ENSG00000177932	protein_coding	82,39064071	233,18966	2,830293076	1,500951	0,035923	1	zinc finger protein 354C [Source:HGNC Symbol;Acc:16736]
CLEC19A	ENSG00000261210	protein_coding	1,745051489	13,25535431	7,595967453	2,925234	0,035951	1	C-type lectin domain family 19, member A [Source:HGNC Symbol;Acc:34522]
FAM203B	ENSG00000230567	protein_coding	0,623376446	9,075358568	14,55839184	3,863779	0,036186	1	family with sequence similarity 203, member B [Source:HGNC Symbol;Acc:32323]
MT1X	ENSG00000187193	protein_coding	47,10632616	16,04031804	0,340513034	-1,55422	0,03631	1	metallothionein 1X [Source:HGNC Symbol;Acc:7405]
FTL	ENSG00000087086	protein_coding	46461,01812	19453,26896	0,418700875	-1,25601	0,036358	1	ferritin, light polypeptide [Source:HGNC Symbol;Acc:3999]
ZNF253	ENSG00000256771	protein_coding	134,0523863	375,3792731	2,800243125	1,485552	0,036425	1	zinc finger protein 253 [Source:HGNC Symbol;Acc:13497]
EFNA2	ENSG00000099617	protein_coding	132,573593	345,2119317	2,6039268	1,380689	0,036557	1	ephrin-A2 [Source:HGNC Symbol;Acc:3222]
LPAR6	ENSG00000139679	protein_coding	217,3326616	530,3485879	2,440261781	1,287036	0,03665	1	lysophosphatidic acid receptor 6 [Source:HGNC Symbol;Acc:15520]
IL7R	ENSG00000168685	protein_coding	22,3104358	5,082266654	0,227797731	-2,13417	0,036712	1	interleukin 7 receptor [Source:HGNC Symbol;Acc:6024]
SULT4A1	ENSG00000130540	protein_coding	138,5773298	389,0883936	2,807734816	1,489407	0,036742	1	sulfotransferase family 4A, member 1 [Source:HGNC Symbol;Acc:14903]
LDHC	ENSG00000166796	protein_coding	6,231751657	0	0	#N/A	0,037193	1	lactate dehydrogenase C [Source:HGNC Symbol;Acc:6544]
PRRT1	ENSG00000204314	protein_coding	77,02436997	187,2011526	2,43041459	1,281202	0,037459	1	proline-rich transmembrane protein 1 [Source:HGNC Symbol;Acc:13943]
SLC8A2	ENSG00000118160	protein_coding	59,06347965	160,9928473	2,725759611	1,446658	0,037612	1	solute carrier family 8 (sodium/calcium exchanger), member 2 [Source:HGNC Symbol;Acc:11069]
RNF148	ENSG00000235631	protein_coding	6,481907358	0	0	#N/A	0,037681	1	ring finger protein 148 [Source:HGNC Symbol;Acc:22411]
HMX1	ENSG00000215612	protein_coding	2,118272234	14,45838219	6,825554318	2,770946	0,038129	1	H6 family homeobox 1 [Source:HGNC Symbol;Acc:5017]
MROH7	ENSG00000184313	protein_coding	15,82651563	50,04954969	3,162385887	1,661013	0,038183	1	maestro heat-like repeat family member 7 [Source:HGNC Symbol;Acc:24802]
MAGEH1	ENSG00000187601	protein_coding	325,6486351	914,0521504	2,806866211	1,48896	0,038239	1	melanoma antigen family H, 1 [Source:HGNC Symbol;Acc:24092]
BEND4	ENSG00000188848	protein_coding	64,80699674	205,5744757	3,172103106	1,66544	0,038683	1	BEN domain containing 4 [Source:HGNC Symbol;Acc:23815]
ELTD1	ENSG00000162618	protein_coding	180,7288493	56,49114621	0,312574038	-1,67773	0,039276	1	EGF, latrophilin and seven transmembrane domain containing 1 [Source:HGNC Symbol;Acc:20822]
MT1E	ENSG00000169715	protein_coding	230,7686084	54,48439964	0,236099702	-2,08253	0,039446	1	metallothionein 1E [Source:HGNC Symbol;Acc:7397]
CD40	ENSG00000101017	protein_coding	9,848945292	83,90247935	8,518930389	3,090672	0,039472	1	CD40 molecule, TNF receptor superfamily member 5 [Source:HGNC Symbol;Acc:11919]
CACNG6	ENSG00000130433	protein_coding	10,09507538	38,70238973	3,833789078	1,938771	0,039526	1	calcium channel, voltage-dependent, gamma subunit 6 [Source:HGNC Symbol;Acc:13625]
TMEM31	ENSG00000179363	protein_coding	2,866726531	15,95196643	5,564523249	2,476258	0,039717	1	transmembrane protein 31 [Source:HGNC Symbol;Acc:28601]
CD74	ENSG00000019582	protein_coding	165,989791	418,8240165	2,52319142	1,33525	0,039722	1	CD74 molecule, major histocompatibility complex, class II invariant chain [Source:HGNC Symbol;Acc:1697]
PSORS1C1	ENSG00000204540	protein_coding	14,08347695	2,390754842	0,169756009	-2,55847	0,03975	1	psoriasis susceptibility 1 candidate 1 [Source:HGNC Symbol;Acc:17202]
DOCK6	ENSG00000130158	protein_coding	530,8812339	1256,820678	2,367423441	1,243318	0,040322	1	dedicator of cytokinesis 6 [Source:HGNC Symbol;Acc:19189]
GRIN3B	ENSG00000116032	protein_coding	7,228348849	27,81228872	3,8476683	1,943984	0,040972	1	glutamate receptor, ionotropic, N-methyl-D-aspartate 3B [Source:HGNC Symbol;Acc:16768]
TAF13	ENSG00000197780	protein_coding	907,5239564	387,2718436	0,426734568	-1,22859	0,04102	1	TAF13 RNA polymerase II, TATA box binding protein (TBP)-associated factor, 18kDa [Source:HGNC Symbol;Acc:11546]
GRID1	ENSG00000182771	protein_coding	179,3429289	69,79750359	0,389184586	-1,36147	0,041057	1	glutamate receptor, ionotropic, delta 1 [Source:HGNC Symbol;Acc:4575]
BEST4	ENSG00000142959	protein_coding	6,231751657	24,33235914	3,904577795	1,965167	0,041234	1	bestrophin 4 [Source:HGNC Symbol;Acc:17106]
HSD11B2	ENSG00000176387	protein_coding	26,04465606	88,67378842	3,404682643	1,76752	0,041661	1	hydroxysteroid (11-beta) dehydrogenase 2 [Source:HGNC Symbol;Acc:5209]
KCNJ15	ENSG00000157551	protein_coding	23,0588901	5,481575845	0,237720715	-2,07266	0,041666	1	potassium inwardly-rectifying channel, subfamily J, member 15 [Source:HGNC Symbol;Acc:6261]
CECR1	ENSG00000093072	protein_coding	205,2967248	621,6918405	3,028259906	1,598489	0,041689	1	cat eye syndrome chromosome region, candidate 1 [Source:HGNC Symbol;Acc:1839]
C8orf47	ENSG00000177459	protein_coding	56,5820507	20,61962297	0,36441986	-1,45633	0,041808	1	chromosome 8 open reading frame 47 [Source:HGNC Symbol;Acc:26823]
WIF1	ENSG00000156076	protein_coding	7,849712488	30,89800942	3,936196322	1,976802	0,0419	1	WNT inhibitory factor 1 [Source:HGNC Symbol;Acc:18081]
PRKG2	ENSG00000138669	protein_coding	15,08208695	43,98176082	2,916158816	1,544069	0,041999	1	protein kinase, cGMP-dependent, type II [Source:HGNC Symbol;Acc:9416]
TMEM74B	ENSG00000125895	protein_coding	59,56580386	152,8656624	2,566332569	1,359708	0,042083	1	transmembrane protein 74B [Source:HGNC Symbol;Acc:15893]
UNC5CL	ENSG00000124602	protein_coding	46,48898813	116,4707762	2,505341177	1,325007	0,042165	1	unc-5 homolog C (C. elegans)-like [Source:HGNC Symbol;Acc:21203]
TYRP1	ENSG00000107165	protein_coding	141,8213027	60,26163215	0,424912415	-1,23476	0,042392	1	tyrosinase-related protein 1 [Source:HGNC Symbol;Acc:12450]
LRRC36	ENSG00000159708	protein_coding	6,604972402	25,23463005	3,820550415	1,93378	0,043126	1	leucine rich repeat containing 36 [Source:HGNC Symbol;Acc:25615]
SNCG	ENSG00000173267	protein_coding	81,75921303	452,5055043	5,534611789	2,468482	0,043346	1	synuclein, gamma (breast cancer-specific protein 1) [Source:HGNC Symbol;Acc:11141]
PCBP3	ENSG00000183570	protein_coding	50,59642913	148,6177163	2,937316305	1,554499	0,043502	1	poly(rC) binding protein 3 [Source:HGNC Symbol;Acc:8651]
PAIP2B	ENSG00000124374	protein_coding	164,002635	434,7710437	2,651000355	1,406537	0,044108	1	poly(A) binding protein interacting protein 2B [Source:HGNC Symbol;Acc:29200]
CPA4	ENSG00000128510	protein_coding	219,2269446	87,93802011	0,401127791	-1,31787	0,044172	1	carboxypeptidase A4 [Source:HGNC Symbol;Acc:15740]
PROB1	ENSG00000228672	protein_coding	27,04125325	94,55977376	3,496870981	1,806065	0,044433	1	proline-rich basic protein 1 [Source:HGNC Symbol;Acc:41906]
KCNQ2	ENSG00000075043	protein_coding	226,3422925	624,1862479	2,757709313	1,46347	0,044531	1	potassium voltage-gated channel, KQT-like subfamily, member 2 [Source:HGNC Symbol;Acc:6296]
IGFLR1	ENSG00000126246	protein_coding	9,346621082	0,798618383	0,085444609	-3,54887	0,044573	1	IGF-like family receptor 1 [Source:HGNC Symbol;Acc:23620]
TAGLN2	ENSG00000158710	protein_coding	2813,856843	1222,136559	0,434327909	-1,20314	0,044954	1	transgelin 2 [Source:HGNC Symbol;Acc:11554]
TTLL6	ENSG00000170703	protein_coding	17,94277506	55,23036856	3,078139718	1,622059	0,045391	1	tubulin tyrosine ligase-like family, member 6 [Source:HGNC Symbol;Acc:26664]
NKD2	ENSG00000145506	protein_coding	123,8865788	284,4269366	2,295865616	1,199038	0,045565	1	naked cuticle homolog 2 (Drosophila) [Source:HGNC Symbol;Acc:17046]
KCNQ4	ENSG00000117013	protein_coding	32,89978416	111,1183544	3,37747974	1,755947	0,045692	1	potassium voltage-gated channel, KQT-like subfamily, member 4 [Source:HGNC Symbol;Acc:6298]
GCKR	ENSG00000084734	protein_coding	4,486700169	19,74285359	4,400305983	2,137604	0,045692	1	glucokinase (hexokinase 4) regulator [Source:HGNC Symbol;Acc:4196]
MT2A	ENSG00000125148	protein_coding	327,5512532	125,2572248	0,382404963	-1,38683	0,045707	1	metallothionein 2A [Source:HGNC Symbol;Acc:7406]
MYH6	ENSG00000197616	protein_coding	382,2916377	97,1968286	0,254247854	-1,97569	0,045737	1	myosin, heavy chain 6, cardiac muscle, alpha [Source:HGNC Symbol;Acc:7576]
CRYAB	ENSG00000109846	protein_coding	141,0849252	57,98983076	0,411027831	-1,28269	0,045777	1	crystallin, alpha B [Source:HGNC Symbol;Acc:2389]
CERS3	ENSG00000154227	protein_coding	20,94061786	3,687234637	0,176080508	-2,50569	0,046034	1	ceramide synthase 3 [Source:HGNC Symbol;Acc:23752]
MYL7	ENSG00000106631	protein_coding	598,7407031	172,7001604	0,288438985	-1,79366	0,046088	1	myosin, light chain 7, regulatory [Source:HGNC Symbol;Acc:21719]
SLITRK4	ENSG00000179542	protein_coding	351,9075002	112,5166792	0,319733678	-1,64506	0,046534	1	SLIT and NTRK-like family, member 4 [Source:HGNC Symbol;Acc:23502]
DMBX1	ENSG00000197587	protein_coding	16,44787927	124,8036198	7,587824406	2,923686	0,046651	1	diencephalon/mesencephalon homeobox 1 [Source:HGNC Symbol;Acc:19026]
FAM184A	ENSG00000111879	protein_coding	233,8067074	529,5346686	2,26483951	1,179409	0,046989	1	family with sequence similarity 184, member A [Source:HGNC Symbol;Acc:20991]
PRSS50	ENSG00000206549	protein_coding	8,226958847	49,28153315	5,990249138	2,582616	0,047898	1	protease, serine, 50 [Source:HGNC Symbol;Acc:17910]
NRK	ENSG00000123572	protein_coding	358,8756293	119,931951	0,334188062	-1,58127	0,047948	1	Nik related kinase [Source:HGNC Symbol;Acc:25391]

CNPY1	ENSG00000146910	protein_coding	5,48329736	23,34173662	4,256879591	2,089796	0,048035	1	canopy FGF signaling regulator 1 [Source:HGNC Symbol;Acc:27786]
CDH15	ENSG00000129910	protein_coding	20,43225523	61,42731149	3,006389201	1,588032	0,048075	1	cadherin 15, type 1, M-cadherin (myotubule) [Source:HGNC Symbol;Acc:1754]
GLRB	ENSG00000109738	protein_coding	93,85352121	212,8299915	2,267682541	1,181219	0,048443	1	glycine receptor, beta [Source:HGNC Symbol;Acc:4329]
TERT	ENSG00000164362	protein_coding	34,14049863	101,4584292	2,971791078	1,571333	0,048474	1	telomerase reverse transcriptase [Source:HGNC Symbol;Acc:11730]
CCDC74B	ENSG00000152076	protein_coding	109,7990763	253,8976345	2,312384067	1,209381	0,048526	1	coiled-coil domain containing 74B [Source:HGNC Symbol;Acc:25267]
ENGASE	ENSG00000167280	protein_coding	537,1371393	1202,625188	2,238953705	1,162825	0,048535	1	endo-beta-N-acetylglucosaminidase [Source:HGNC Symbol;Acc:24622]
ATP8A1	ENSG00000124406	protein_coding	391,6865661	983,9381667	2,512054923	1,328868	0,048634	1	ATPase, aminophospholipid transporter (APLT), class I, type 8A, member 1 [Source:HGNC Symbol;Acc:13531]
ROBO1	ENSG00000169855	protein_coding	2163,604478	4790,523607	2,21414018	1,146747	0,048824	1	roundabout, axon guidance receptor, homolog 1 (Drosophila) [Source:HGNC Symbol;Acc:10249]
SYNPO	ENSG00000171992	protein_coding	1820,720982	858,8502539	0,471708879	-1,08403	0,048855	1	synaptopodin [Source:HGNC Symbol;Acc:30672]
UBE2T	ENSG00000077152	protein_coding	259,0968707	594,2566523	2,293569392	1,197595	0,04907	1	ubiquitin-conjugating enzyme E2T (putative) [Source:HGNC Symbol;Acc:25009]
TEKT3	ENSG00000125409	protein_coding	13,33703546	38,5987372	2,894101715	1,533116	0,049187	1	tektin 3 [Source:HGNC Symbol;Acc:14293]
CHRDL1	ENSG00000101938	protein_coding	87,72873215	210,0450278	2,394255823	1,259577	0,049274	1	chordin-like 1 [Source:HGNC Symbol;Acc:29861]
HIF3A	ENSG00000124440	protein_coding	346,0690973	803,6865116	2,322329609	1,215573	0,049347	1	hypoxia inducible factor 3, alpha subunit [Source:HGNC Symbol;Acc:15825]
MT1F	ENSG00000198417	protein_coding	79,87901966	31,89373225	0,399275459	-1,32454	0,049414	1	metallothionein 1F [Source:HGNC Symbol;Acc:7398]
PON1	ENSG00000005421	protein_coding	16,07868414	3,386477667	0,21061908	-2,24729	0,049638	1	paraoxonase 1 [Source:HGNC Symbol;Acc:9204]
ANGPTL1	ENSG00000116194	protein_coding	32,40349837	9,671772202	0,29847926	-1,7443	0,049974	1	angiopoietin-like 1 [Source:HGNC Symbol;Acc:489]
ANKRD20A11P	ENSG00000215559	pseudogene	106,7951951	0	0	#N/A	6,88E-13	9,09E-09	ankyrin repeat domain 20 family, member A11, pseudogene [Source:HGNC Symbol;Acc:42024]
PSPHP1	ENSG00000226278	pseudogene	2,118272234	157,8085744	74,49872206	6,219144	3,93E-11	3,11E-07	phosphoserine phosphatase pseudogene 1 [Source:HGNC Symbol;Acc:9578]
RPSAP53	ENSG00000214263	pseudogene	75,1461894	4,78660999	0,063697308	-3,97262	4,09E-06	0,013505	ribosomal protein SA pseudogene 53 [Source:HGNC Symbol;Acc:36641]
CYP4F29P	ENSG00000228314	pseudogene	437,1105216	1,892893429	0,004330469	-7,85126	6,12E-06	0,018659	cytochrome P450, family 4, subfamily F, polypeptide 29, pseudogene [Source:HGNC Symbol;Acc:2647]
DDX11L2	ENSG00000236397	pseudogene	134,6070435	18,04706461	0,134072216	-2,89892	8,38E-05	0,132775	DEAD/H (Asp-Glu-Ala-Asp/His) box helicase 11 like 2 [Source:HGNC Symbol;Acc:37103]
RP11-262H14.7	ENSG00000227582	pseudogene	0	21,03933339	#N/A	#N/A	0,000105	0,15349	
RP11-578O24.2	ENSG00000254352	pseudogene	0,623376446	25,42663419	40,78857059	5,350093	0,000131	0,185498	
RP11-259G18.3	ENSG00000262539	pseudogene	3,114869425	43,16784152	13,85863599	3,792713	0,000175	0,231458	
DND1P1	ENSG00000264070	pseudogene	8,848322486	71,38453974	8,067578894	3,012136	0,00023	0,293984	dead end homolog 1 pseudogene 1 [Source:HGNC Symbol;Acc:31444]
HERC2P3	ENSG00000180229	pseudogene	47,96174304	286,409828	5,971630927	2,578125	0,000275	0,330325	hect domain and RLD 2 pseudogene 3 [Source:HGNC Symbol;Acc:4871]
AC018865.9	ENSG00000231240	pseudogene	14,45065928	100,0990994	6,926957273	2,792222	0,000434	0,43118	
CTD-2514C3.1	ENSG00000204556	pseudogene	0	16,75058481	#N/A	#N/A	0,000508	0,47714	
AC018865.8	ENSG00000180178	pseudogene	297,8796233	1397,562609	4,691702622	2,230112	0,000694	0,61087	
RP11-217H19.1	ENSG00000215104	pseudogene	0	12,16617957	#N/A	#N/A	0,003099	1	
OR7E101P	ENSG00000227019	pseudogene	0,623376446	24,93897339	40,00628117	5,322155	0,003509	1	olfactory receptor, family 7, subfamily E, member 101 pseudogene [Source:HGNC Symbol;Acc:15342]
OR2A9P	ENSG00000228960	pseudogene	10,71845183	0	0	#N/A	0,003784	1	olfactory receptor, family 2, subfamily A, member 9 pseudogene [Source:HGNC Symbol;Acc:8236]
AC069213.1	ENSG00000224769	pseudogene	0	11,66831816	#N/A	#N/A	0,003788	1	
USP32P2	ENSG00000233327	pseudogene	5,858530912	33,20551296	5,667890715	2,502812	0,006153	1	ubiquitin specific peptidase 32 pseudogene 2 [Source:HGNC Symbol;Acc:30751]
RP11-823P9.3	ENSG00000233974	pseudogene	2,866726531	23,23298379	8,104359987	3,018698	0,006187	1	
ZNF385C	ENSG00000187595	protein_coding	41,50197656	145,8905023	3,515266366	1,813634	0,006379	1	zinc finger protein 385C [Source:HGNC Symbol;Acc:33722]
ENPP7P10	ENSG00000249767	pseudogene	0	10,5638425	#N/A	#N/A	0,007091	1	ectonucleotide pyrophosphatase/phosphodiesterase 7 pseudogene 10 [Source:HGNC Symbol;Acc:48693]
NOL5BP	ENSG00000235559	pseudogene	2,368427935	20,43781945	8,62927647	3,10924	0,007589	1	
SNX18P13	ENSG00000230965	pseudogene	8,848322486	0	0	#N/A	0,008911	1	sorting nexin 18 pseudogene 13 [Source:HGNC Symbol;Acc:39621]
RP11-334A14.2	ENSG00000236360	pseudogene	10,59136117	0,399309191	0,037701404	-4,72924	0,011197	1	
MED15P9	ENSG00000223760	pseudogene	7,474478936	39,27330183	5,254319687	2,393504	0,012085	1	mediator complex subunit 15 pseudogene 9 [Source:HGNC Symbol;Acc:44130]
FER1L4	ENSG00000088340	pseudogene	118,9136569	566,0519622	4,760193041	2,25102	0,012816	1	fer-1-like 4 (C. elegans), pseudogene [Source:HGNC Symbol;Acc:15801]
AC023085.1	ENSG00000235661	pseudogene	1,745051489	16,2527234	9,313606794	3,21934	0,01315	1	
AACSP1	ENSG00000250420	pseudogene	0	8,774601598	#N/A	#N/A	0,014364	1	acetoacetyl-CoA synthetase pseudogene 1 [Source:HGNC Symbol;Acc:18226]
FNBP1P1	ENSG00000257800	pseudogene	59,81193394	9,982729785	0,166901973	-2,58293	0,014946	1	formin binding protein 1 pseudogene 1 [Source:HGNC Symbol;Acc:44528]
CRHR1-IT1	ENSG00000204650	pseudogene	365,6721022	979,6102621	2,678930813	1,421657	0,015999	1	CRHR1 intronic transcript 1 (non-protein coding) [Source:HGNC Symbol;Acc:26327]
RP11-208G20.2	ENSG00000204894	pseudogene	6,730050253	32,09593699	4,769048638	2,253701	0,01604	1	
RP11-551L14.1	ENSG00000177359	pseudogene	8,848322486	37,20370518	4,204605476	2,07197	0,016166	1	
SEC1P	ENSG00000232871	pseudogene	1,745051489	14,96134391	8,573583077	3,099898	0,01797	1	secretory blood group 1, pseudogene [Source:HGNC Symbol;Acc:44149]
MYO5BP2	ENSG00000238245	pseudogene	1,494895787	14,85769138	9,938947926	3,313093	0,020299	1	myosin VB pseudogene 2 [Source:HGNC Symbol;Acc:38496]
SPDYE6	ENSG00000166667	pseudogene	19,19355357	3,386477667	0,176438285	-2,50276	0,021231	1	speedy/RINGO cell cycle regulator family member E6 [Source:HGNC Symbol;Acc:35465]
AL133458.1	ENSG00000197146	pseudogene	9,346621082	36,80439599	3,937722057	1,977361	0,021449	1	
RP11-216M21.1	ENSG00000237846	pseudogene	18,69324216	3,386477667	0,18116053	-2,46466	0,021585	1	
RP11-566K19.6	ENSG00000259344	pseudogene	0,996597191	12,66404098	12,70728143	3,667584	0,021624	1	
GTF2H2B	ENSG00000226259	pseudogene	55,70449294	158,1042311	2,838267125	1,50501	0,026552	1	general transcription factor IIH, polypeptide 2B (pseudogene) [Source:HGNC Symbol;Acc:31393]
FTLP3	ENSG00000226608	pseudogene	933,9881278	368,4890107	0,394532864	-1,34178	0,027057	1	ferritin, light polypeptide pseudogene 3 [Source:HGNC Symbol;Acc:4000]
RP11-195E2.1	ENSG00000230847	pseudogene	26,42190242	78,36990044	2,966096052	1,568565	0,027394	1	
AP001065.7	ENSG00000224747	pseudogene	4,111466617	21,73939955	5,28750482	2,402587	0,02984	1	
COL6A4P2	ENSG00000228252	pseudogene	139,2047318	350,1633979	2,515456143	1,33082	0,030013	1	collagen, type VI, alpha 4 pseudogene 2 [Source:HGNC Symbol;Acc:38501]
PI4KAP1	ENSG00000215513	pseudogene	727,6766905	1800,130449	2,473805293	1,306732	0,031809	1	phosphatidylinositol 4-kinase, catalytic, alpha pseudogene 1 [Source:HGNC Symbol;Acc:33576]
UBE2FP1	ENSG00000224080	pseudogene	29,16757671	3,588682416	0,123036701	-3,02284	0,032912	1	ubiquitin-conjugating enzyme E2F (putative) pseudogene 1 [Source:HGNC Symbol;Acc:44535]
RP11-707O23.5	ENSG00000263503	pseudogene	11,34384108	37,18840426	3,278290308	1,712944	0,033303	1	
AC010984.3	ENSG00000230646	pseudogene	0	6,98026039	#N/A	#N/A	0,033434	1	
RP11-1166P10.1	ENSG00000260628	pseudogene	18,69122936	4,283648271	0,229179589	-2,12545	0,036006	1	
AC108463.2	ENSG00000227992	pseudogene	33,65025126	9,666671895	0,287268937	-1,79953	0,036314	1	
RP11-22B23.1	ENSG00000111788	pseudogene	171,2309839	421,3779811	2,460874613	1,299171	0,036371	1	
TAGLN2P1	ENSG00000253676	pseudogene	268,4656327	105,7675791	0,393970647	-1,34384	0,037602	1	transgelin 2 pseudogene 1 [Source:HGNC Symbol;Acc:21739]
DUTP1	ENSG00000229048	pseudogene	5,981595956	0	0	#N/A	0,039016	1	deoxyuridine triphosphatase pseudogene 1 [Source:HGNC Symbol;Acc:31956]
RANP4	ENSG00000225125	pseudogene	10,71643902	37,58771345	3,507481673	1,810436	0,039215	1	RAN, member RAS oncogene family pseudogene 4 [Source:HGNC Symbol;Acc:39859]
RP11-255H23.2	ENSG00000233836	pseudogene	19,31058019	62,0033239	3,210847281	1,682954	0,041954	1	
FTLP2	ENSG00000232368	pseudogene	354,9435863	149,2157763	0,420392936	-1,25019	0,043308	1	ferritin, light polypeptide pseudogene 2 [Source:HGNC Symbol;Acc:4001]
ZNF204P	ENSG00000204789	pseudogene	228,0672159	529,3069623	2,320837567	1,214646	0,044052	1	zinc finger protein 204, pseudogene [Source:HGNC Symbol;Acc:12995]
OR7E38P	ENSG00000183444	pseudogene	73,89943651	28,69929871	0,388355583	-1,36455	0,044932	1	olfactory receptor, family 7, subfamily E, member 38 pseudogene [Source:HGNC Symbol;Acc:8411]
KRT18P24	ENSG00000226234	pseudogene	7,476491743	0,497861413	0,066590244	-3,90855	0,046051	1	keratin 18 pseudogene 24 [Source:HGNC Symbol;Acc:33393]
SPATA31C1	ENSG00000230246	pseudogene	0,498298596	10,47549089	21,02251738	4,393864	0,046365	1	SPATA31 subfamily C, member 1 [Source:HGNC Symbol;Acc:27846]
KRT18P36	ENSG00000226246	pseudogene	7,351413892	0,497861413	0,067723219	-3,88421	0,046807	1	keratin 18 pseudogene 36 [Source:HGNC Symbol;Acc:33405]
RP11-706O15.5	ENSG00000205663	lincRNA	3,114869425	164,1295711	52,69227973	5,71952	1,54E-10	1,02E-06	
AC104135.3	ENSG00000204792	lincRNA	92,83679595	1,493584238	0,016088279	-5,95785	7,87E-10	4,46E-06	
RP11-138B4.1	ENSG00000250658	lincRNA	0,498298596	73,98259964	148,4704157	7,214032	4,57E-09	2,27E-05	
RP11-11N5.1	ENSG00000250829	lincRNA	0	46,1601103	#N/A	#N/A	1,04E-07	0,000456	

SNORD116-20	ENSG00000261069	lincRNA	68,79338551	6,876607862	0,099960306	-3,3225	5,47E-05	0,108307	small nucleolar RNA, C/D box 116-20 [Source:HGNC Symbol;Acc:33086]
RP11-262H14.3	ENSG00000234665	lincRNA	0	19,44719693	#N/A	#N/A	0,000175	0,231458	
LINC00152	ENSG00000222041	lincRNA	233,2963319	56,49624652	0,242165173	-2,04594	0,001308	0,942256	long intergenic non-protein coding RNA 152 [Source:HGNC Symbol;Acc:28717]
MIR4435-1HG	ENSG00000172965	lincRNA	351,4494577	93,16638814	0,265091853	-1,91544	0,002068	1	MIR4435-1 host gene (non-protein coding) [Source:HGNC Symbol;Acc:35163]
RP13-977J11.2	ENSG00000256576	lincRNA	0,623376446	18,03686399	28,93414421	4,854701	0,002449	1	
RP11-545G3.1	ENSG00000241449	lincRNA	1,246752893	22,54311824	18,08146456	4,17644	0,002625	1	
KLHL7-AS1	ENSG00000230658	lincRNA	7,726647444	45,26804	5,858691021	2,550578	0,003336	1	KLHL7 antisense RNA 1 (head to head) [Source:HGNC Symbol;Acc:43431]
RP11-262H14.4	ENSG00000170161	lincRNA	5,235154466	32,5054468	6,209071196	2,634377	0,004997	1	
LINC00890	ENSG00000260802	lincRNA	194,4048878	62,84949143	0,323291725	-1,62909	0,007016	1	long intergenic non-protein coding RNA 890 [Source:HGNC Symbol;Acc:48576]
RP4-610C12.4	ENSG00000205611	lincRNA	17,06924291	73,59349106	4,311467793	2,108179	0,007191	1	
RP11-1055B8.4	ENSG00000262877	lincRNA	123,997567	377,7887828	3,046743511	1,607268	0,009964	1	
LINC00565	ENSG00000260910	lincRNA	24,79991597	4,086543829	0,164780551	-2,60138	0,010192	1	long intergenic non-protein coding RNA 565 [Source:HGNC Symbol;Acc:43709]
LRCOL1	ENSG00000204583	lincRNA	3,986388766	41,1967971	10,33436514	3,369378	0,01108	1	leucine rich colipase-like 1 [Source:HGNC Symbol;Acc:44160]
RP11-187O7.3	ENSG00000259124	lincRNA	0	9,676872508	#N/A	#N/A	0,014098	1	
RP11-189B4.6	ENSG00000231817	lincRNA	24,3076558	289,7097616	11,91845746	3,575126	0,016913	1	
RP11-689P11.2	ENSG00000205959	lincRNA	31,28584894	94,4306197	3,018317319	1,593744	0,017737	1	
CTB-175P5.4	ENSG00000269416	lincRNA	160,8917912	440,2916145	2,736569785	1,452369	0,019412	1	
RP11-426L16.8	ENSG00000215866	lincRNA	1,619973638	14,06417331	8,681729738	3,117983	0,023844	1	
ZNF667-AS1	ENSG00000166770	lincRNA	70,260102	245,6225407	3,495903561	1,805665	0,02678	1	ZNF667 antisense RNA 1 (head to head) [Source:HGNC Symbol;Acc:44321]
CTD-2545M3.8	ENSG00000268518	lincRNA	53,70526014	9,780525036	0,182114843	-2,45708	0,027016	1	
RP11-315I14.2	ENSG00000233906	lincRNA	8,598166785	0,399309191	0,046441201	-4,42845	0,028426	1	
RP11-168O16.1	ENSG00000229191	lincRNA	66,66504924	179,8895334	2,698408468	1,432109	0,033072	1	
RP4-669L17.10	ENSG00000237094	lincRNA	12,83873687	40,97929143	3,191847598	1,674392	0,034646	1	
RP11-470L19.2	ENSG00000235407	lincRNA	13,83130844	2,292202621	0,165725653	-2,59313	0,035226	1	
RP11-509J21.3	ENSG00000235326	lincRNA	5,858530912	33,41791832	5,704146453	2,512011	0,035825	1	
RP11-66D17.3	ENSG00000237588	lincRNA	5,110076615	22,04015652	4,31307751	2,108718	0,036417	1	
RP11-809C9.2	ENSG00000258102	lincRNA	3,613168021	37,41101024	10,35407433	3,372127	0,036892	1	
LINC00312	ENSG00000237697	lincRNA	220,7178148	89,89376362	0,407279148	-1,29591	0,038309	1	long intergenic non-protein coding RNA 312 [Source:HGNC Symbol;Acc:6662]
KBTBD11-OT1	ENSG00000253696	lincRNA	7,353426699	27,42318015	3,72930625	1,898907	0,039333	1	KBTBD11 overlapping transcript 1 (non-protein coding) [Source:HGNC Symbol;Acc:49147]
AC159540.1	ENSG00000230606	lincRNA	203,1402092	83,31791272	0,410149783	-1,28578	0,039507	1	
RP11-63P12.6	ENSG00000225434	lincRNA	10,84151687	1,395032017	0,128674985	-2,9582	0,041528	1	
AC069513.4	ENSG00000229178	lincRNA	4,486700169	20,72837581	4,619960111	2,20788	0,042882	1	
RP4-760C5.3	ENSG00000231081	lincRNA	0	6,383846756	#N/A	#N/A	0,043153	1	
RP11-662J14.2	ENSG00000258480	lincRNA	0	6,285294535	#N/A	#N/A	0,044267	1	
RP11-849I19.1	ENSG00000263146	lincRNA	43,37411871	106,1193391	2,446605078	1,290781	0,044949	1	
AP000997.2	ENSG00000260254	lincRNA	0,996597191	10,56894281	10,60502969	3,406677	0,045229	1	
RP11-420G6.4	ENSG00000230438	lincRNA	4,984998764	24,11485347	4,837484343	2,274257	0,047108	1	
RP4-610C12.3	ENSG00000225490	lincRNA	6,853115297	26,03324844	3,798746601	1,925523	0,047732	1	
RP11-108M12.3	ENSG00000258592	lincRNA	15,08208695	2,592959591	0,17192313	-2,54016	0,048513	1	
RP11-195E2.4	ENSG00000254353	lincRNA	50,48141532	129,1603187	2,558571663	1,355339	0,048549	1	
CTD-2127H9.1	ENSG00000249740	lincRNA	5,733453061	0	0	#N/A	0,049935	1	
HOTAIRM1	ENSG00000233429	antisense	227,7141232	1003,611262	4,407329894	2,139905	0,000435	0,43118	HOXA transcript antisense RNA, myeloid-specific 1 [Source:HGNC Symbol;Acc:37117]
MRPL23-AS1	ENSG00000226416	antisense	16,69803497	88,06717417	5,274104067	2,398926	0,002215	1	MRPL23 antisense RNA 1 [Source:HGNC Symbol;Acc:42812]
NPPA-AS1	ENSG00000242349	antisense	85,74962741	19,22969126	0,224253934	-2,15679	0,002959	1	NPPA antisense RNA 1 [Source:HGNC Symbol;Acc:37635]
RP11-54O7.11	ENSG00000224969	antisense	7,476491743	45,07093556	6,028353553	2,591764	0,003403	1	
TMEM254-AS1	ENSG00000230091	antisense	211,1290892	57,1283623	0,270584989	-1,88585	0,004812	1	TMEM254 antisense RNA 1 [Source:HGNC Symbol;Acc:27340]
RP11-421F16.3	ENSG00000247903	antisense	78,52127856	251,7770348	3,206481599	1,680991	0,007308	1	
RP11-1060J15.4	ENSG00000256377	antisense	19,81693001	2,095098178	0,105722641	-3,24164	0,008078	1	
FAM85B	ENSG00000253893	antisense	2,368427935	19,73775329	8,333693838	3,058956	0,00942	1	family with sequence similarity 85, member B [Source:HGNC Symbol;Acc:32160]
AC116614.1	ENSG00000235688	antisense	4,486700169	32,71785216	7,292186001	2,866351	0,012655	1	
DICER1-AS1	ENSG00000235706	antisense	102,8208831	294,3009136	2,862267903	1,517159	0,013776	1	DICER1 antisense RNA 1 [Source:HGNC Symbol;Acc:43017]
RP11-796E2.4	ENSG00000245904	antisense	12,96180191	54,26014727	4,186157731	2,065627	0,015084	1	
RP11-909N17.2	ENSG00000253931	antisense	7,974790339	35,80357286	4,489594251	2,166585	0,016514	1	
CTC-559E9.8	ENSG00000267565	antisense	11,33780266	45,55859636	4,018291527	2,006582	0,022124	1	
ACTA2-AS1	ENSG00000180139	antisense	27,79172035	6,378746449	0,229519669	-2,12331	0,023156	1	ACTA2 antisense RNA 1 [Source:HGNC Symbol;Acc:45169]
CTD-2314G24.2	ENSG00000259663	antisense	19,81089159	67,00233925	3,382096103	1,757918	0,025564	1	
KANSL1-AS1	ENSG00000214401	antisense	9,471698933	36,38468557	3,841410694	1,941636	0,02715	1	KANSL1 antisense RNA 1 [Source:HGNC Symbol;Acc:43740]
FOXD2-AS1	ENSG00000237424	antisense	24,54774746	76,48720762	3,115854428	1,639628	0,027361	1	FOXD2 antisense RNA 1 (head to head) [Source:HGNC Symbol;Acc:44256]
RP11-685N10.1	ENSG00000255893	antisense	21,8101244	4,584405241	0,210196199	-2,25019	0,028488	1	
MYLK-AS2	ENSG00000250174	antisense	0	7,182465139	#N/A	#N/A	0,028668	1	MYLK antisense RNA 2 [Source:HGNC Symbol;Acc:40387]
AC007277.3	ENSG00000213981	antisense	4,859920914	22,73002207	4,677035383	2,225594	0,035446	1	
ST7-AS1	ENSG00000227199	antisense	30,90256416	88,34242961	2,858741079	1,51538	0,038221	1	ST7 antisense RNA 1 [Source:HGNC Symbol;Acc:16000]
RP11-524D16__A.3	ENSG00000261295	antisense	20,44030646	4,682957462	0,229104073	-2,12592	0,040699	1	
RP11-142C4.6	ENSG00000255240	antisense	9,596776783	32,11633822	3,346575517	1,742686	0,041737	1	
AC108488.3	ENSG00000234171	antisense	193,0370826	79,05466568	0,40953098	-1,28796	0,041788	1	
RP4-758J18.7	ENSG00000225905	antisense	0,498298596	8,478944934	17,01579135	4,088802	0,045387	1	
RP11-617D20.1	ENSG00000231160	antisense	48,10292335	161,2919579	3,353059371	1,745478	0,048987	1	
RP11-494M8.4	ENSG00000254951	sense_overlapping	10,46829612	0	0	#N/A	0,003942	1	
CTD-2240H23.2	ENSG00000259143	sense_overlapping	38,75227665	108,5576431	2,801322979	1,486108	0,04081	1	
RP11-150O12.6	ENSG00000253414	sense_intronic	22,5605915	2,489307063	0,110338732	-3,17999	0,010024	1	
RP11-31F19.1	ENSG00000226403	sense_intronic	13,83533406	2,193650399	0,158554206	-2,65695	0,046606	1	
RN7SL128P	ENSG00000240869	misc_RNA	6,106673806	30,11979226	4,932274626	2,302253	0,01411	1	RNA, 7SL, cytoplasmic 128, pseudogene [Source:HGNC Symbol;Acc:46144]
RN7SL4P	ENSG00000263740	misc_RNA	31,28383614	97,03888021	3,10188558	1,633145	0,01567	1	RNA, 7SL, cytoplasmic 4, pseudogene [Source:HGNC Symbol;Acc:10039]
SLC38A3	ENSG00000188338	processed_transcript	43,61823599	135,6019153	3,10883538	1,636374	0,015535	1	solute carrier family 38, member 3 [Source:HGNC Symbol;Acc:18044]
GRIP2	ENSG00000144596	processed_transcript	183,8437187	445,1071799	2,421117148	1,275673	0,028549	1	glutamate receptor interacting protein 2 [Source:HGNC Symbol;Acc:23841]
FAM182A	ENSG00000125804	processed_transcript	0,996597191	40,11272266	40,24968463	5,330906	0,045056	1	family with sequence similarity 182, member A [Source:HGNC Symbol;Acc:16222]
MIR3188	ENSG00000267959	miRNA	6,106673806	29,99573851	4,91196017	2,296299	0,02318	1	microRNA 3188 [Source:HGNC Symbol;Acc:38226]
ACTN3	ENSG00000248746	polymorphic_pseudogene	161,1157804	1782,135032	11,06120721	3,467437	0,034359	1	actinin, alpha 3 (gene/pseudogene) [Source:HGNC Symbol;Acc:165]

Table S3. Mean expression levels of selected transcripts

Gene_Name	Ensembl_GeneID	Norm_Expr_CTR	Norm_Expr_PD	Fold Change	Log2FC	p-Value	FDR
NRK	ENSG00000123572	358,8756293	119,931951	0,334188062	-1,58127	0,047948	1
GABRQ	ENSG00000147402	24,29960457	90,56158154	3,726874702	1,897966	0,010639	1
SYNPO	ENSG00000171992	1820,720982	858,8502539	0,471708879	-1,08403	0,048855	1
GRID1	ENSG00000182771	179,3429289	69,79750359	0,389184586	-1,36147	0,041057	1
ROBO1	ENSG00000169855	2163,604478	4790,523607	2,21414018	1,146747	0,048824	1
SYT7	ENSG00000011347	153,4173123	437,9637097	2,854721565	1,51335	0,015105	1
FLRT2	ENSG00000185070	4883,326574	2112,195619	0,432532125	-1,20912	0,028656	1
CA7	ENSG00000168748	0,996597191	17,24334592	17,30222207	4,112885	0,005405	1
TYW3	ENSG00000162623	821,2679792	2,292202621	0,002791053	-8,48497	5,56E-21	2,2E-16
CRYZ	ENSG00000116791	2082,900517	23,60679145	0,011333614	-6,46325	8,7E-18	1,72E-13
NEUROD1	ENSG00000162992	30,91061539	255,4473221	8,264064591	3,046852	0,00271	1
NEUROG1	ENSG00000181965	27,91881101	225,2493789	8,068014745	3,012214	0,017308	1
NEUROG2	ENSG00000178403	60,31627096	160,2299311	2,656495976	1,409525	0,033535	1

Table S4: Metabolism genes differentially expressed in PD NPCs

Gene Name	Ensembl ID	Fold Change	P Value
TYW3	ENSG00000162623	-358.2876888	5.55695E-21
CRYZ	ENSG00000116791	-88.23310536	8.69542E-18
ALOX15B	ENSG00000179593	-10.96044941	0.002391622
GLIPR1	ENSG00000139278	-5.789328554	0.008093524
CERS3	ENSG00000154227	-5.679220316	0.046034028
NPPA	ENSG00000175206	-4.81276838	0.021750623
PON1	ENSG00000005421	-4.747907922	0.049637821
NNMT	ENSG00000166741	-4.712467017	0.003097069
AGPAT9	ENSG00000138678	-4.56615704	0.00234267
PTGR1	ENSG00000106853	-3.880024692	0.001216553
NQO1	ENSG00000181019	-3.601718303	0.01461021
GBP1	ENSG00000117228	-3.496958894	0.011255777
ADM	ENSG00000148926	-3.314921756	0.006712054
MT1X	ENSG00000187193	-2.936745147	0.036310079
SGMS2	ENSG00000164023	-2.811229932	0.01449496
C8orf47	ENSG00000177459	-2.744087551	0.041808012
GCLM	ENSG00000023909	-2.646676561	0.017963359
MT2A	ENSG00000125148	-2.615028823	0.045706868
ME1	ENSG00000065833	-2.552089519	0.018529803
GBP2	ENSG00000162645	-2.515165983	0.032366983
MT1F	ENSG00000198417	-2.504536598	0.049413631
FTL	ENSG00000087086	-2.388339884	0.036358478
TYRP1	ENSG00000107165	-2.353426179	0.042392464
RARRES2	ENSG00000106538	2.436248962	0.03247455
LPAR6	ENSG00000139679	2.440261781	0.036650243
SLC25A27	ENSG00000153291	2.479652827	0.027245583
ATP8A1	ENSG00000124406	2.512054923	0.048633584
ADSSL1	ENSG00000185100	2.557635784	0.032336745
ALPL	ENSG00000162551	2.622445675	0.023642969
LOXL3	ENSG00000115318	2.635309735	0.031876936
PLCG2	ENSG00000197943	2.651948423	0.033817974
SULT4A1	ENSG00000130540	2.807734816	0.036742355
CHST6	ENSG00000183196	2.898144984	0.034269525
PCBP3	ENSG00000183570	2.937316305	0.043502172
TXNRD2	ENSG00000184470	3.081689229	0.019533308
GLYATL2	ENSG00000156689	3.13347574	0.027466881
PTGS1	ENSG00000095303	3.312172748	0.014517288
HSD11B2	ENSG00000176387	3.404682643	0.041661168
CLEC18C	ENSG00000157335	3.455942627	0.035127595
FGF19	ENSG00000162344	3.587461678	0.00642645
SELV	ENSG00000186838	3.844732878	0.016689422
MPPED1	ENSG00000186732	3.987888575	0.026383513
OAS3	ENSG00000111331	4.31083521	0.016880971
GCKR	ENSG00000084734	4.400305983	0.045692123

LDHD	ENSG00000166816	5.408713699	0.005267177
CRIP3	ENSG00000146215	6.129611905	0.002701273
SLC25A41	ENSG00000181240	6.327857186	0.000369596
IAH1	ENSG00000134330	6.568371109	0.003128166
CLEC19A	ENSG00000261210	7.595967453	0.03595115
CA7	ENSG00000168748	17.30222207	0.005404687

Table S5: Differentiation and development genes differentially expressed in PD NPCs

Gene Name	Ensembl ID	Fold Change	P Value
GDF3	ENSG00000184344	-19.7784609	0.020208572
OSGIN1	ENSG00000140961	-4.254543149	0.004522714
OGN	ENSG00000106809	-3.64454594	0.030626325
NELL1	ENSG00000165973	-3.054963518	0.007379073
EDN1	ENSG00000078401	-3.040441542	0.023771623
GBP4	ENSG00000162654	-2.569345675	0.027054243
YPEL1	ENSG00000100027	2.388812142	0.033124823
CHRDL1	ENSG00000101938	2.394255823	0.049273684
ERBB3	ENSG00000065361	2.491644739	0.022412989
EFNA2	ENSG00000099617	2.6039268	0.036556641
SOX8	ENSG00000005513	2.608236077	0.026364651
NEUROG2	ENSG00000178403	2.656495976	0.033535267
CECR1	ENSG00000093072	3.028259906	0.041688725
DTX1	ENSG00000135144	3.13293344	0.025339592
HEYL	ENSG00000163909	3.242285646	0.009112501
EYA2	ENSG00000064655	3.303160502	0.016748188
LAPTM5	ENSG00000162511	3.710498843	0.026363452
POU4F1	ENSG00000152192	3.771616001	0.002346524
WIF1	ENSG00000156076	3.936196322	0.041899595
LGI4	ENSG00000153902	4.031282321	0.001981302
NTRK1	ENSG00000198400	4.306336836	0.002167139
FOXD3	ENSG00000187140	4.436183895	0.031795119
ISLR2	ENSG00000167178	4.43665464	0.03120838
DCDC2	ENSG00000146038	4.74519173	0.001158024
FOXN4	ENSG00000139445	5.121896246	0.002378932
C1orf111	ENSG00000171722	5.327106125	0.025977285
NTN5	ENSG00000142233	5.450933318	0.023027582
NOTUM	ENSG00000185269	5.619951989	0.000402492
CRHR1	ENSG00000120088	6.356920716	0.001353505
NEUROG1	ENSG00000181965	8.068014745	0.017307738
MAATS1	ENSG00000183833	8.116360465	0.033476156
NEUROD1	ENSG00000162992	8.264064591	0.002709899

Table S6: Ion Channels and transporters genes differentially expressed in PD NPCs

Gene Name	Ensembl ID	Fold Change	P Value
BEST3	ENSG00000127325	-7.592920769	0.007234843
KCNE4	ENSG00000152049	-6.062222787	0.028434074
KCNJ15	ENSG00000157551	-4.206616993	0.041666306
KCNAB3	ENSG00000170049	2.476934922	0.03173247
KCNQ2	ENSG00000075043	2.757709313	0.044531124
KCNC1	ENSG00000129159	2.779455197	0.021826856
SLC38A3	ENSG00000188338	3.10883538	0.015534549
KCNQ4	ENSG00000117013	3.37747974	0.045691928
KCNK5	ENSG00000164626	3.432348566	0.005808151
CACNG6	ENSG00000130433	3.833789078	0.039525831
BEST4	ENSG00000142959	3.904577795	0.041233713
KCNQ1	ENSG00000053918	4.110293874	0.015587053
KCNH6	ENSG00000173826	7.910264198	0.005683123

Table S7: Cytoskeleton genes differentially expressed in PD NPCs

Gene Name	Ensembl ID	Fold Change	P Value
ACTG2	ENSG00000163017	-9.512168282	2.70154E-07
TAGLN	ENSG00000149591	-5.195740077	7.89406E-05
UPK1B	ENSG00000114638	-5.164231465	0.004127269
PALMD	ENSG00000099260	-4.1395118	0.002380045
ACTA2	ENSG00000107796	-4.018529289	0.000789845
MYH6	ENSG00000197616	-3.93316987	0.045736589
SYNM	ENSG00000182253	-3.335647775	0.008319921
PDLIM3	ENSG00000154553	-3.247964637	0.017971558
NRK	ENSG00000123572	-2.992327119	0.047948388
MAP7D3	ENSG00000129680	-2.649763506	0.021541752
MYOM2	ENSG00000036448	-2.535778723	0.034178157
SYNPO	ENSG00000171992	-2.119951614	0.048854522
THSD7B	ENSG00000144229	2.760013134	0.019674973
TEKT3	ENSG00000125409	2.894101715	0.049186939
TTLL6	ENSG00000170703	3.078139718	0.045391137
DNAH2	ENSG00000183914	3.325797689	0.006786459
JAKMIP1	ENSG00000152969	5.20252008	0.0221527
SAMSN1	ENSG00000155307	13.67514708	0.003892129

Table S8: Cell adhesion and ECM genes differentially expressed in PD NPCs

**GENE_NAME**	**Gene_ID**	**foldChange_Easy**	**PValue**
CTHRC1	ENSG00000164932	-9.821087576	7.32935E-05
ZP3	ENSG00000188372	-7.586842856	0.001262508
SPP1	ENSG00000118785	-4.126368165	0.000806018
TNC	ENSG00000041982	-3.806591353	0.001039882
TNFRSF12A	ENSG00000006327	-3.752375047	0.009520479
ADAMTS16	ENSG00000145536	-3.087351233	0.017423181
SERPINB8	ENSG00000166401	-2.926149315	0.019458065
LAMC2	ENSG00000058085	-2.880063533	0.011048531
CRYAB	ENSG00000109846	-2.43292528	0.045777113
TAGLN2	ENSG00000158710	-2.302407879	0.044954293
FAM184A	ENSG00000111879	2.26483951	0.046989494
NKD2	ENSG00000145506	2.295865616	0.04556459
COL9A2	ENSG00000049089	2.310379664	0.035700122
CDH23	ENSG00000107736	2.82314754	0.019080159
FLRT1	ENSG00000126500	2.842617557	0.032092894
CDH15	ENSG00000129910	3.006389201	0.04807502
ITGA4	ENSG00000115232	3.165026731	0.021486217
NXPH3	ENSG00000182575	3.222671774	0.031451081
ADAMTS4	ENSG00000158859	3.324561207	0.014936464
CGREF1	ENSG00000138028	3.368264776	0.029006964
CDHR2	ENSG00000074276	3.374616153	0.01331885
ITGA2B	ENSG00000005961	5.661234457	0.000263149
COL11A2	ENSG00000204248	6.116583841	0.005391696
ART5	ENSG00000167311	6.490506413	0.000426201
PCDHA6	ENSG00000081842	14.90708	2.62655E-07

Table S9: Synapse genes differentially expressed in PD NPCs

**Gene Name**	**Ensembl ID**	**Fold Change**	**P Value**
SLITRK4	ENSG00000179542	-3.12760297	0.046533977
GRID1	ENSG00000182771	-2.569474833	0.041057084
FLRT2	ENSG00000185070	-2.311967003	0.028655985
ROBO1	ENSG00000169855	2.21414018	0.048823714
GLRB	ENSG00000109738	2.267682541	0.04844259
GRIP2	ENSG00000144596	2.421117148	0.02854949
PRRT1	ENSG00000204314	2.43041459	0.037458522
CHRNB4	ENSG00000117971	2.704193211	0.028629695
SYCP2L	ENSG00000153157	2.8506323	0.021811882
SYT7	ENSG00000011347	2.854721565	0.015104993
PRKG2	ENSG00000138669	2.916158816	0.041998645
DOC2A	ENSG00000149927	2.98289427	0.028019024
CADM2	ENSG00000175161	3.079221913	0.012164982
GRIK4	ENSG00000149403	3.244705989	0.018435817
GABRQ	ENSG00000147402	3.726874702	0.0106387
GRIN3B	ENSG00000116032	3.8476683	0.040971722
LY6H	ENSG00000176956	4.103052055	0.001162307
SYNGR3	ENSG00000127561	4.817786337	0.030258958
C1orf173	ENSG00000178965	4.861641392	0.02970214
SNCG	ENSG00000173267	5.534611789	0.043346414
TULP1	ENSG00000112041	6.486963414	0.001649509
COMT	ENSG00000093010	8.873079174	0.000335146
SLC6A3	ENSG00000142319	12.83009295	0.028470159

Table S10: Disease associated genes differentially expressed in PD NPCs

Gene Name	Ensembl ID	Fold Change	Disease-associated
TYW3	ENSG00000162623	0,003	ALS
CRYZ	ENSG00000116791	0,011	ALS
GDF3	ENSG00000184344	0,051	Klippel-Feil syndrome 3, autosomal dominant (KFS3); Microphthalmia, isolated, with coloboma, 6
CAV1	ENSG00000105974	0,349	Lipodystrophy
GLRB	ENSG00000109738	2,268	Hyperekplexia 2 (HKPX2)
TEKT3	ENSG00000125409	2,894	Charcot-Marie-Tooth disease
CHST6	ENSG00000183196	2,898	Macular dystrophy
PRKG2	ENSG00000138669	2,916	Epilepsy, Temporal lobe epilepsy, Intellectual disability
PCBP3	ENSG00000183570	2,937	Nutritional and Metabolic Diseases
CERKL	ENSG00000188452	2,970	Retinitis pigmentosa 26
CDH15	ENSG00000129910	3,006	Mental retardation
CADM2	ENSG00000175161	3,079	Mental disorders
CDK18	ENSG00000117266	3,097	Alzheimer's disease
CAMK1G	ENSG00000008118	3,208	ALS
NXPH3	ENSG00000182575	3,223	Sjögren-Larsson syndrome
ADAMTS4	ENSG00000158859	3,325	Alzheimer's disease
IGLON5	ENSG00000142549	3,614	Tauopathies, Encephalitis, Movement disorders, Cerebellar Ataxia
GFRA2	ENSG00000168546	3,664	Parkinsonian disorders, Schizophrenia, Bipolar disorder, FTD, Neuropathy
GABRQ	ENSG00000147402	3,727	Schizophrenia, ASD, Anxiety disorders
PDYN	ENSG00000101327	3,771	Spinocerebellar ataxia 23 (SCA23), Mental depression, Mood disorders, Schizophrenia, Bipolar disorder

GRIN3B	ENSG00000116032	3,848	Schizophrenia, Alzheimer's disease; ALS, Epilepsy
LGI4	ENSG00000153902	4,031	Arthrogryposis multiplex congenita, neurogenic, with myelin defect (AMCNMY)
NTRK1	ENSG00000198400	4,306	Congenital insensitivity to pain with anhidrosis (CIPA)
TMEM132E	ENSG00000181291	4,637	patient with 17q11.2 microdeletion syndrome (facial dysmorphism, mental retardation, developmental delay, and an excessive number of neurofibromas)
SYNGR3	ENSG00000127561	4,818	Alzheimer's disease
C1orf173	ENSG00000178965	4,862	Mental disorders
CALN1	ENSG00000183166	5,592	Schizophrenia
PRDM12	ENSG00000130711	6,193	Neuropathy, hereditary sensory and autonomic, 8 (HSAN8)
HMX1	ENSG00000215612	6,826	Oculoauricular syndrome (OCACS)1
NEUROG1	ENSG00000181965	8,068	Mental disorders
NEUROD1	ENSG00000162992	8,264	Maturity-onset diabetes of the young 6 (MODY6)
COMT	ENSG00000093010	8,873	Schizophrenia
SLC6A3	ENSG00000142319	12,830	Parkinsonism-dystonia infantile (PKDYS)

Table S11: DEGs mean expression in iPSC-derived NPC and neurons

GENE NAME	Mean Expression at NPC stage	Mean Expression at Neuron stage	P value
HES1	1497	875	0,060216
NFIA	183,5	1798	0,000204
LMX1A	858,5	147,25	0,035347
LMX1B	859	941,75	0,86885
OTX2	1294,25	266,25	0,017262
FOXA2	49,25	26	0,281164
DCX	1603,75	10856,25	0,010774
MAP2	4479	16271	0,005457
TUBB3	132,25	449,25	0,008913
RBFOX3	71,75	498,5	0,00571
TH	77,25	842	0,030418
KCNJ6	11	40,25	0,102857
PBX1	4601	5372	0,394065
LMO3	626,75	929,75	0,285857
SYT1	795,75	2701	0,004246
SNAP25	386,25	1962,5	0,0012

Table S12: Metabolism genes expression in PD neurons

Gene Name	Ensembl ID	Fold Change	P Value
**TYW3**	**ENSG00000162623**	**-7,73042002**	**4,3351E-16**
**CRYZ**	**ENSG00000116791**	**-9,35666627**	**1,6354E-27**
ALOX15B	ENSG00000179593	0	1
GLIPR1	ENSG00000139278	-0,11454588	0,87792097
CERS3	ENSG00000154227	-0,95825583	0,50271101
NPPA	ENSG00000175206	-0,47878303	0,3580525
PON1	ENSG00000005421	0,34094006	1
**NNMT**	**ENSG00000166741**	**-1,86371017**	**0,0446333**
AGPAT9	ENSG00000138678	0,10319326	0,87069888
**PTGR1**	**ENSG00000106853**	**-1,94022541**	**0,00021669**
NQO1	ENSG00000181019	0,30455055	0,4522212
GBP1	ENSG00000117228	-0,40409305	0,64022594
ADM	ENSG00000148926	-0,92650638	0,16769368
MT1X	ENSG00000187193	-0,22701909	0,95529553
SGMS2	ENSG00000164023	0,84281495	0,12462623
C8orf47	ENSG00000177459	-0,38117838	0,94409111
GCLM	ENSG00000023909	0,00861202	0,94607204
MT2A	ENSG00000125148	-0,10546017	0,9884541
ME1	ENSG00000065833	-0,51860901	0,27232623
GBP2	ENSG00000162645	1,21184794	0,72558748
MT1F	ENSG00000198417	1,42962666	0,48979961
FTL	ENSG00000087086	-0,08971125	0,68288573
TYRP1	ENSG00000107165	-0,80225254	0,54950897
RARRES2	ENSG00000106538	-0,66558143	0,70105019
LPAR6	ENSG00000139679	0,64545913	0,34079903
SLC25A27	ENSG00000153291	-0,11880006	0,82930883
ATP8A1	ENSG00000124406	-0,00059872	0,78429645
ADSSL1	ENSG00000185100	-0,38172422	0,40459282
**ALPL**	**ENSG00000162551**	**1,67562119**	**0,00527182**
LOXL3	ENSG00000115318	0,16283252	0,76874227
PLCG2	ENSG00000197943	1,49545168	0,06346525
SULT4A1	ENSG00000130540	-0,5043528	0,429242
**CHST6**	**ENSG00000183196**	**-1,58864916**	**0,00217305**
PCBP3	ENSG00000183570	0,09405466	0,77525366
**TXNRD2**	**ENSG00000184470**	**2,4313034**	**0,01038325**
GLYATL2	ENSG00000156689	0,45524892	0,36675665
**PTGS1**	**ENSG00000095303**	**1,29783299**	**0,04638282**
HSD11B2	ENSG00000176387	-0,16600545	0,86875626
CLEC18C	ENSG00000157335	1,31105665	0,11381725

FGF19	ENSG00000162344	1,71508199	0,13615063
SELV	ENSG00000186838	1,50750235	0,10607643
MPPED1	ENSG00000186732	-0,35530487	0,64885027
OAS3	ENSG00000111331	0,0359823	0,98994395
GCKR	ENSG00000084734	-0,83818735	0,46109736
LDHD	ENSG00000166816	-0,93276758	0,10899141
CRIP3	ENSG00000146215	-0,32138131	0,69911775
SLC25A41	ENSG00000181240	0,61275162	0,33802711
**IAH1**	**ENSG00000134330**	**1,90184679**	**0,0121563**
CLEC19A	ENSG00000261210	-0,01152898	0,88379615
CA7	ENSG00000168748	-0,39468628	0,71878321

Table S13: Differentiation and Development genes expression in PD neurons

Gene Name	Ensembl ID	Fold Change	P Value
GDF3	ENSG00000184344	-1,01523754	0,436831113
**OSGIN1**	**ENSG00000140961**	**-1,32659683**	**0,007057145**
OGN	ENSG00000106809	0,415353047	1
NELL1	ENSG00000165973	-0,35459829	0,83492534
EDN1	ENSG00000078401	0,418083383	0,470813774
GBP4	ENSG00000162654	-1,55950408	0,352716138
YPEL1	ENSG00000100027	-0,05616904	0,852711898
CHRDL1	ENSG00000101938	-0,58557981	0,302794299
ERBB3	ENSG00000065361	-0,71393394	0,140150876
EFNA2	ENSG00000099617	-0,1529949	0,804873787
SOX8	ENSG00000005513	-0,80340381	0,069688058
NEUROG2	ENSG00000178403	0,679385941	0,416547903
**CECR1**	**ENSG00000093072**	**2,162941506**	**0,000757315**
DTX1	ENSG00000135144	-0,8022317	0,325358431
HEYL	ENSG00000163909	-0,01106932	0,977214465
**EYA2**	**ENSG00000064655**	**1,67480419**	**0,003458066**
LAPTM5	ENSG00000162511	-0,23648863	0,62600918
POU4F1	ENSG00000152192	0,242671535	0,484081309
WIF1	ENSG00000156076	0,157309374	0,88997811
LGI4	ENSG00000153902	-0,48019045	0,402845352
NTRK1	ENSG00000198400	1,81266908	0,066607888
FOXD3	ENSG00000187140	-0,24955468	0,969716191
ISLR2	ENSG00000167178	-0,65545087	0,22986653
DCDC2	ENSG00000146038	#N/A	0,562016675
FOXN4	ENSG00000139445	0,311774187	0,47150559
C1orf111	ENSG00000171722	0,090358105	0,855553271
NTN5	ENSG00000142233	-0,28200946	0,535558961

NOTUM	ENSG00000185269	-0,16464993	0,830160869
CRHR1	ENSG00000120088	-0,51293854	0,23484302
NEUROG1	ENSG00000181965	0,03709293	0,854716792
MAATS1	ENSG00000183833	1,956619734	0,065780305
NEUROD1	ENSG00000162992	0,602480959	0,396867729

Table S14: Ion Channels & Transporters genes expression in PD neurons

**Gene Name**	**Ensembl ID**	**Fold Change**	**P Value**
BEST3	ENSG00000127325	1,226453273	0,564078453
KCNE4	ENSG00000152049	0,925214234	0,842844698
KCNJ15	ENSG00000157551	1,032442307	0,849315545
KCNAB3	ENSG00000170049	1,020376415	0,96878023
KCNQ2	ENSG00000075043	0,975483991	0,749588277
KCNC1	ENSG00000129159	1,580912598	0,309223942
SLC38A3	ENSG00000188338	1,187557438	0,613165883
KCNQ4	ENSG00000117013	0,754387631	0,864047388
KCNK5	ENSG00000164626	0,60556424	0,282371088
CACNG6	ENSG00000130433	0,964890978	0,937560238
BEST4	ENSG00000142959	0,917782831	0,669284561
KCNQ1	ENSG00000053918	2,089425442	0,063488186
KCNH6	ENSG00000173826	1,768795027	0,415431368

Table S15: Cell Adhesion & ECM genes expression in PD neurons

**GENE_NAME**	**ENSEMBL ID**	**Fold Change**	**P Value**
**CTHRC1**	**ENSG00000164932**	**-2,792084341**	**0,049278235**
**ZP3**	**ENSG00000188372**	**-1,931154232**	**0,005079308**
SPP1	ENSG00000118785	-1,385216646	0,269432328
TNC	ENSG00000041982	-0,237561242	0,651553299
TNFRSF12A	ENSG00000006327	-0,596322387	0,186048066
ADAMTS16	ENSG00000145536	-0,094442651	0,778898814
SERPINB8	ENSG00000166401	-0,258077166	0,714920437
LAMC2	ENSG00000058085	-1,04806624	0,119306128
CRYAB	ENSG00000109846	-0,459884204	0,452134393
TAGLN2	ENSG00000158710	-0,710732326	0,116919659
FAM184A	ENSG00000111879	0,09754491	0,816672328
NKD2	ENSG00000145506	0,07037187	0,81246326
COL9A2	ENSG00000049089	0,507811369	0,301747253
CDH23	ENSG00000107736	-0,1855838	0,768193455
FLRT1	ENSG00000126500	-0,691714365	0,113143354
**CDH15**	**ENSG00000129910**	**-1,006192761**	**0,036483845**
ITGA4	ENSG00000115232	-0,16366983	0,655521856

NXPH3	ENSG00000182575	-1,713792158	0,31671285
ADAMTS4	ENSG00000158859	-0,130056827	0,691678416
CGREF1	ENSG00000138028	-0,139841559	0,939851222
CDHR2	ENSG00000074276	0,443634141	0,470659809
ITGA2B	ENSG00000005961	-0,362171928	0,436176836
COL11A2	ENSG00000204248	0,33721123	0,804420597
**ART5**	**ENSG00000167311**	**1,94357123**	**0,013448789**
**PCDHA6**	**ENSG00000081842**	**2,26000165**	**0,00519631**

Table S16: Synapse-associated genes expression in PD neurons

**Gene Name**	**Ensembl ID**	**Fold Change**	**P Value**
**SLITRK4**	**ENSG00000179542**	**-2,096086518**	**5,53298E-05**
GRID1	ENSG00000182771	-0,969066527	0,087599644
FLRT2	ENSG00000185070	0,023361819	0,796876513
ROBO1	ENSG00000169855	-0,024305202	0,801483468
GLRB	ENSG00000109738	0,009257127	0,956742668
**GRIP2**	**ENSG00000144596**	**-1,211083365**	**0,015363659**
PRRT1	ENSG00000204314	-0,247679307	0,500570141
CHRNB4	ENSG00000117971	-0,133523119	1
SYCP2L	ENSG00000153157	-0,195332404	0,507502306
SYT7	ENSG00000011347	-0,893415622	0,137854816
PRKG2	ENSG00000138669	0,336601587	0,622624745
DOC2A	ENSG00000149927	-0,748039137	0,528130378
CADM2	ENSG00000175161	-0,487076773	0,690029003
GRIK4	ENSG00000149403	-0,900717154	0,088316529
GABRQ	ENSG00000147402	-0,896692823	0,15011532
GRIN3B	ENSG00000116032	0,134416584	0,971023085
LY6H	ENSG00000176956	0,625875237	0,246297842
SYNGR3	ENSG00000127561	-1,02924112	0,112523322
C1orf173	ENSG00000178965	1,110223798	0,441980185
SNCG	ENSG00000173267	0,034025394	0,972734622
TULP1	ENSG00000112041	1,485300948	0,218735106
**COMT**	**ENSG00000093010**	**4,05315194**	**0,003152408**
SLC6A3	ENSG00000142319	0,789603819	0,603926287

Table S17: Cytoskeleton genes expression in PD neurons

**Gene Name**	**Ensembl ID**	**Fold Change**	**P Value**
ACTG2	ENSG00000163017	-0,293354527	0,650390595
TAGLN	ENSG00000149591	-0,335685968	0,458114991
UPK1B	ENSG00000114638	0,284587507	0,994074939
PALMD	ENSG00000099260	-0,145112743	0,685605657

ACTA2	ENSG00000107796	0,142994441	0,811931362
MYH6	ENSG00000197616	0,410769592	0,979210371
SYNM	ENSG00000182253	-0,11481957	0,767161477
PDLIM3	ENSG00000154553	-0,524632155	0,266939459
NRK	ENSG00000123572	-0,458731002	0,223838342
**MAP7D3**	**ENSG00000129680**	**-1,959574414**	**0,008451711**
MYOM2	ENSG00000036448	-0,203715774	0,62576461
SYNPO	ENSG00000171992	-0,030267254	0,784157213
THSD7B	ENSG00000144229	0,472749606	0,495638921
TEKT3	ENSG00000125409	0,100438087	0,992808474
TTLL6	ENSG00000170703	0,069561816	0,869873967
DNAH2	ENSG00000183914	-0,418971938	0,410745351
JAKMIP1	ENSG00000152969	-1,160509345	0,325821723
SAMSN1	ENSG00000155307	1,285389794	0,362205922
